# BAP1 Represses Sequential Activation of IRAKs and NF-κB Signaling in Pancreatic Cancer

**DOI:** 10.7150/ijbs.104977

**Published:** 2025-02-18

**Authors:** Yuhan Zhao, Xueyi Liang, Ruozheng Wei, Feng Guo, Gengdu Qin, Haixin Yu, Jiaying Liu, Wentao Xia, Shanmiao Gou, Heshui Wu, Yingke Zhou

**Affiliations:** 1 Department of Pancreatic Surgery, Union Hospital, Tongji Medical College, Huazhong University of Science and Technology, Wuhan, 430022 Hubei China.; 2 Sino-German Laboratory of Personalized Medicine for Pancreatic Cancer, Union Hospital, Tongji Medical College, Huazhong University of Science and Technology, Wuhan, 430022 China.; 3 Cancer Center, Union Hospital, Tongji Medical College, Huazhong University of Science and Technology, Wuhan, 430022 China.; 4 Department of Pathology, Union Hospital, Tongji Medical College, Huazhong University of Science and Technology, Wuhan, China.

## Abstract

The deubiquitinating enzyme BRCA1 Associated Protein-1 (BAP1) has been reported to be shallowly deleted in a subset of pancreatic ductal adenocarcinomas (PDAC) and is believed to play a significant role in the development of chronic pancreatitis-driven PDAC. However, evidence suggests that BAP1 may also be involved in the progression and metastasis of PDAC, though the underlying mechanism remains unclear. Here, we demonstrate that BAP1 deletion leads to the overactivation of the nuclear factor-κB (NF-κB) signaling in PDAC, thereby promoting the proliferation, migration, and invasion of PDAC models both* in vivo* and* in vitro*. Mechanistically, BAP1 inhibits the sequential activation of interleukin-1 receptor-associated kinases (IRAKs) in an enzyme-independent manner. BAP1 binds to IRAK1 and inhibits the interaction between IRAK4 and IRAK1, as well as the IRAK4-mediated initiation of IRAK1 phosphorylation and autophosphorylation. This, in turn, prevents the dissociation of IRAK1 from the Myddosome complex and sequential activation of NF-κB. Based on this, we further identified that dual-target inhibitors of IRAK1/4 exhibited significant inhibitory effects on BAP1-deficient tumors in both *in vivo* and *in vitro* PDAC models. Our findings elucidate the mechanism by which BAP1 inhibits the NF-κB signaling and present a promising strategy for the targeted treatment of BAP1-deficient pancreatic cancer.

## Introduction

Pancreatic cancer poses a significant and escalating global public health concern due to its rising incidence, limited therapeutic options and dismal prognosis^1^, ^2^. In the past decade, several large-scale unbiased sequencing studies of PDAC have confirmed intertumoural and intratumoural heterogeneity at the transcriptional level may contribute to dramatically different clinical outcomes of cancers that behave morphologically similar^3^-^6^. To explore more about specific genetic alterations may be beneficial to identify the subtypes of PDAC susceptible to targeted therapies.

BAP1 is a member of the deubiquitinating enzyme class of proteins. Its discovery dates back to 1998, where it was initially described as a nuclear protein exhibiting specific binding affinity for the RING finger domain of the BRCA1 protein^7^, ^8^. Subsequent studies suggested BAP1 as an independent tumor suppressor^9^, ^10^. Carriers of germline BAP1 mutations often suffer multiple cancers during their lifetime, a condition was named the “BAP1 cancer syndrome”. BAP1 and its associated proteins play an important role in a multitude of biological processes such as DNA damage, cell cycle, metabolic homeostasis, and ferroptosis. It's been reported frequently heterozygous loss of BAP1 in pancreatic cancer. The loss of BAP1 leads to genomic instability, which contribute to chronic pancreatitis and predisposes to cancer development^11^. Previous studies also investigated that BAP1 links ferroptosis to tumor suppression by repressing SLC7A11 expression through BAP1-mediated H2Aub deubiquitination on SLC7A11 in PDAC cells^12^. Furthermore, the Hippo pathway was deregulated in BAP1-deficient PDAC as negative regulator of the pathway, LATS2, was ubiquitinated and degraded^13^. However, the mechanism by which BAP1 regulates tumorigenesis, the signaling pathways that are variation in BAP1-deficient tumors, and the potential therapeutic strategy for BAP1-deficient tumors remain largely unexplored.

The nuclear factor-κB (NF-κB) transcription factor family is the pivotal participant in innate and adaptive immune responses. Epidemiological studies have identified chronic inflammation as a key risk factor for various types of cancer, including pancreatic cancer. NF-κB pathway links chronic inflammation to the cancer^14^, ^15^, and could also regulate tumor angiogenesis^16^, invasiveness^17^ and many other sections in tumorigenesis and therapy^18^-^20^. Targeting NF-κB or the signaling pathway that activate it may be a potentially effective strategy. BAP1 has been reported to inactivate the NF-κB signaling through enhancing stability of BRCA1 protein in sepsis-induced acute kidney injury model^21^. Previous study also proved BAP1 is pertaining to NF-κB pathway, and link the chronic pancreatitis to cancer^11^. Nevertheless, the specific mechanism of how BAP1 interplay with the activation of NF-κB remains unclear.

Members of the IRAK family are key molecules in the signaling cascade of the Toll/IL-1 receptor (TIR) family, playing a crucial role in the induction of inflammatory molecule expression in cancer cells. It is currently understood that, within this family, only IRAK1 and IRAK4 exhibit enzymatic activity^22^. IRAK1 exists initially in an inactive state in the cytoplasm and is recruited by activated TLR/IL-1R receptors, where it binds to MyD88 and IRAK4 to form the IRAK complex. Phosphorylation of IRAK1 by IRAK4 is essential for the subsequent propagation of caspase signals. Notably, constitutive activation of IRAK4 has been linked to resistance to checkpoint immunotherapy and chemotherapy, thereby contributing to poor prognosis in pancreatic ductal adenocarcinoma^23^-^25^. Targeting the IRAK1/4 signaling pathway has emerged as a promising therapeutic strategy for various malignancies, including diffuse large B-cell lymphoma (DLBCL), myelodysplastic syndrome, breast cancer, head and neck cancer, and pancreatic cancer. However, the mechanisms underlying IRAK1/4 activation in PDAC and the identification of patient subsets that would benefit from IRAK1/4 inhibition remain elusive.

In this study, we demonstrated that BAP1 deletion facilitates the progression and metastasis of PDAC. We presented multi-level evidence showing that BAP1 inhibits NF-κB signaling in PDAC. We elaborated on the specific role of BAP1 in the sequential activation of IRAKs and established that BAP1-deficient PDAC exhibits increased sensitivity to IRAK1/4-targeted inhibition.

## Materials and methods

### Cell culture and transfection

As previously described, all cell lines utilized in this study (293T, CFPAC-1, PaTu8988, SW1990, and BxPC-3) were maintained in Dulbecco's Modified Eagle Medium (DMEM) (basal media, China) supplemented with 10% fetal bovine serum (FBS). The cells were cultured at 37°C in a 5% CO₂ incubator. Routine certification procedures were employed to ensure cell line purity and the absence of infections. For transfection, 1-2 μL of Lipofectamine 8000 was used per 1 μg of plasmid DNA. Lipofectamine 8000 and plasmid DNA were diluted separately in Opti-MEM and subsequently mixed. The resulting DNA-lipid complex was incubated at room temperature for 15 minutes before being added to the cells.

### RNAi

The lentiviral packaging vectors psPAX2 and pMD2.G, along with the specific shRNA constructs, were co-transfected into 293T cells. 24 hours post-transfection, the culture medium was replaced with fresh DMEM supplemented with 10% FBS. 72 hours after transfection, the supernatant containing the lentiviral particles was collected and used to infect cancer cells in the presence of polybrene (12 μg/mL). Selection with puromycin was initiated 48 hours post-infection, using concentrations ranging from 3 to 5 μg/mL. The lentivirus-based small hairpin RNAs (shRNAs) were provided as a gift by Zhou (Mayo Clinic). The sequence information for the shRNAs is detailed in the Supplementary Table.

### Coimmunoprecipitation and Western blot analysis

As previously described, cells were treated with immunoprecipitation (IP) lysis buffer (1 mL per 10^7 cells; Biosharp) on ice for 30 minutes, supplemented with 1 mmol/L phenylmethylsulfonyl fluoride (PMSF; Beyotime Biotechnology) and a phosphatase inhibitor (Beyotime Biotechnology). The cell lysate was then centrifuged at 14,000 g at 4°C for 10 minutes. The supernatant was subsequently incubated with primary antibody and protein A/G agarose beads (Beyotime Biotechnology) at 4°C overnight. The beads were washed on ice with ice-cold IP lysis buffer (Beyotime Biotechnology) five times, and then harvested for Western blot analysis.

Protein concentration was determined using a bicinchoninic acid (BCA) protein assay. Equal amounts of denatured proteins were separated by SDS-PAGE and transferred to a nitrocellulose membrane. The membrane was blocked with 1× TBST containing 5% BSA at room temperature for 1 hour. Specific primary antibodies were then applied to the membrane and incubated at 4°C overnight. Following incubation, the membrane was washed three times with 1× TBST. The membrane was then incubated with specific secondary antibodies at room temperature for 1 hour. Protein detection was performed using a super-sensitive electrochemiluminescence reagent.

### Immunofluorescent chemistry

Cells were seeded at a density of 40,000 cells per well in 12-well plates containing cell slides (Beijing, China). After allowing the cells to adhere, they were fixed with 1 mL of paraformaldehyde per well for 10 minutes, followed by two washes with PBS. Next, 600 μL of 0.25% Triton X-100 (Solarbio, Beijing, China) was added to each well for 5-8 minutes, after which the cells were washed three times with PBS. Blocking was performed using goat serum (BOSTER, Wuhan, China), and after two additional washes with PBS, the corresponding diluted primary antibody was added and incubated overnight at 4°C.

Following primary antibody incubation, the cells were washed three times with PBS and then exposed to 1 mL of FITC/Cy3-conjugated goat anti-rabbit/mouse IgG (Proteintech, China) in the dark for 1 hour. After incubation, the cells were washed three times with PBS, and 300 μL of DAPI staining solution (Proteintech, China) was added to each well for 4 minutes. After three additional washes with PBS, the slides were mounted with anti-fade mounting medium (Proteintech, China) and observed using a confocal microscope.

Fluorescence quantitation is conducted utilizing the IMAGEJ software. In essence, the delineation of cytoplasmic and nuclear regions is achieved through the utilization of DAPI staining, enabling the precise measurement of the average fluorescence intensity within the designated nuclear and cytoplasmic regions. Following this, an adjacent cellular area is selected for the purpose of calculating the background signal. Ultimately, the relative nuclear/cytoplasmic fluorescence intensity is derived by subtracting the background signal from the previously ascertained average intensity.

### Nuclear and cytoplasmic protein extraction

Nuclear and cytoplasmic proteins were extracted using the Nuclear and Cytoplasmic Protein Extraction Kit (Beyotime, China) following the manufacturer's instructions. The resulting extracts were then utilized for Western blot or co-immunoprecipitation (Co-IP) analysis.

### Colony formation assay

A total of 1,000 tumor cells infected with indicated plasmids, with or without IRAK1/4 inhibitor treatment, were plated in six-well plates. Colony formation assays were conducted, and colonies were photographed after 7 to 12 days of plating.

### Cell invasion assay

Cell invasion ability was assessed using transwell chambers with 8 μm pores. Tumor cells were seeded in the Matrigel-coated upper chamber of the inserts, and DMEM supplemented with 30% FBS was placed in the lower chamber. The setup was incubated for 24 hours at 37°C with 5% CO2. After incubation, cells were fixed in methanol for 20 minutes and subsequently stained with Crystal Violet stain solution (#C0121, Beyotime). Invasive cells were quantified by microscopy, examining five fields per well. All experimental assays were conducted in triplicate.

### Wound-healing assay

Pancreatic cancer cells were plated in 6-well plates and cultured for 24 hours. Following this, a scratch was made in the center of each well using a 10 μL pipette tip, and medium without FBS was added. Wound healing was assessed as the percentage change in wound size over the healing period. All assays were performed in triplicate.

### Quantitative RT-PCR

RNA was extracted from cancer cells using TRIzol (Thermo Fisher Scientific). Two micrograms of RNA were used to synthesize cDNA following the instructions provided with the PrimeScript™ RT Reagent Kit (Tsingke, China). Quantitative PCR was performed using the TB Green™ Fast qPCR Mix (Tsingke, China). The fold change in gene expression was calculated using the '2^-ΔΔC(T)' method, with GAPDH as the reference gene for normalization. Primers with amplification efficiencies ranging from 90% to 110% were employed for quantitative analysis. The sequences of the primers used for RT-qPCR are listed in the Supplementary Table.

### Tissue microarray and IHC

The tissue microarray (TMA) slides were provided by OUTDO BIOTECH(Shanghai). These slides were subjected to immunostaining using the respective specific antibodies. The staining intensity was evaluated and scored according to previously established criteria. The median scores for BAP1 and p-p65 across all samples were used as the cut-off values. The assessment was carried out by two independent pathologists who were blinded to the experimental conditions.

### Chromatin immunoprecipitation (ChIP) and ChIP-qPCR

Chromatin immunoprecipitation (ChIP) was performed using the Chromatin Extraction Kit (Abcam, USA) and the ChIP Kit Magnetic - One Step (Abcam, USA). The purified DNA obtained from the ChIP procedure was analyzed using methods similar to those employed for RT-qPCR. The specific primers used for ChIP-qPCR are detailed in the Supplementary Table.

### *In vivo* tumor models

Four-week-old nude mice(male), procured from Vital River Laboratories in Beijing, China, and KPC (LSL-KrasG12D/+; LSL-Trp53R172H/+; Pdx-1-Cre, 8 weeks old, sex-matched) transgenic mice and Bap1 knock-out KPC mice were purchased from MODEL ORGANISMS Inc. (Shanghai, China) were utilized in this investigation. Ethical approval for the study was obtained from the Ethics Committee of Tongji Medical College, Huazhong University of Science and Technology. Subcutaneously, WT/BAP1 knockout PaTu8988 cells, totaling 5 × 10^6 in 100 µL of 1 × PBS, were injected into mice via tail vein injection. These groups were administered either IRAK1/4 inhibitor (Sigma I5409, intraperitoneal injections daily at 4mg/kg) or PBS treatment for two weeks. On day 30, mice were euthanized, with exceptions made for those meeting euthanasia criteria prior to the endpoint due to unforeseen events such as infection. All experimental procedures involving mice strictly adhered to the guidelines set forth by the local ethics committee of Tongji Medical College, Huazhong University of Science and Technology, China.

### Dual-luciferase reporter gene assay

PDAC cells were suspended at a concentration of 2 × 10^4 cells per 0.1 mL and seeded into a 96-well plate. Following this, they were transfected with reporter plasmids (Catalog No. LVG00115Z, Creative Biogene) at a concentration of 100 ng per well, along with the appropriate expression plasmids, according to the protocols detailed in the cell culture and transfection section. A pRL-TK vector (Promega), which encodes Renilla luciferase, was used as an internal control to assess transfection efficiency. Luciferase activities in each well were quantitatively measured using the Dual-Luciferase Reporter Assay System (Promega).

### Bioinformatics mining

The correlation between BAP1/IRAK1 and the prognosis of TCGA pancreatic cancer patients was analyzed using the Gene Expression Profiling Interactive Analysis (GEPIA) database (http://gepia.cancer-pku.cn/). The CNV and expression of BAP1 was analyzed using the cbioportal (https://www.cbioportal.org/). Differentially expressed genes (DEGs) in the TCGA pancreatic cancer dataset and GSE16515/GSE55643/GSE120078 dataset were identified using R (version 4.1.0) and DESeq2 package (http://bioconductor.org/packages/release/bioc/html/DESeq2.html). KEGG pathway enrichment analysis and Gene Set Enrichment Analysis (GSEA) were performed using the clusterProfiler package (version 4.0), Enrichr (https://maayanlab.cloud/Enrichr/) and gene signatures from MSigDB/ChEA.

### Statistical analysis

Statistical analyses were conducted using GraphPad Prism 9 software (GraphPad Software, Inc). Statistical significance was determined using Student's t-test, and one-way or two-way ANOVA, followed by Tukey's multiple comparisons test. A p-value of less than 0.05 was considered significant. All data are expressed as the mean ± SD.

## Result

### BAP1 loss promotes the proliferation, migration and invasion of PDAC cells

**Figure 1:**
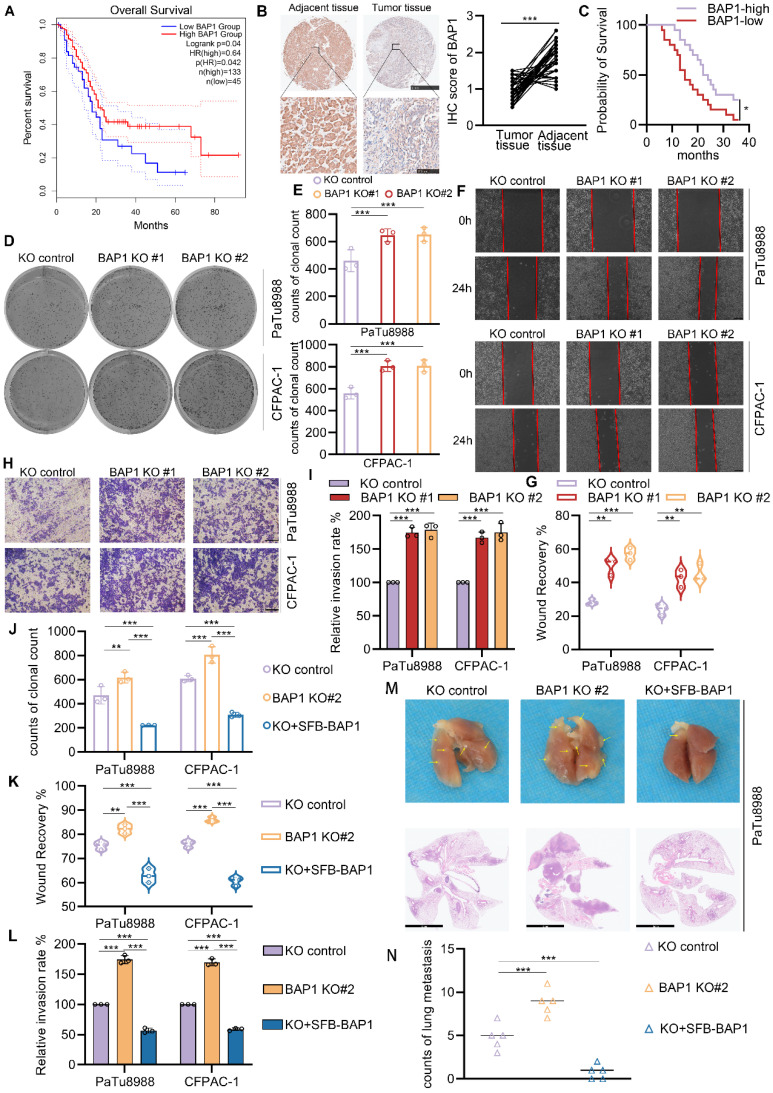
** BAP1 loss promotes the proliferation, migration and invasion of PDAC cells. A**, Kaplan-Meier plots showing the overall survival between BAP1 high and low patients in TCGA-PAAD dataset. **B**, Represent images of the IHC analysis of the indicated proteins within pancreatic cancer tissues and their adjacent tissues in patients diagnosed with pancreatic cancer (left) and the quantitative data (right). n = 40, two-tailed paired Student's t-test. **C**, Kaplan-Meier plots showing the overall survival between BAP1 high and low patients in in-house tumor cohort. **D-E**, After the infection of indicated lentivirus and puromycin selection, 10 days after plate operation, the clonogenicity of PaTu8988 and CFPAC-1 cells were measured (D) and quantified (E). n=3. **F-G**, After the infection of indicated lentivirus and puromycin selection, PaTu8988 and CFPAC-1 cells were harvested for wound-healing assay (F) and the quantitative data (G). n=3. **H-I**, After the infection of indicated lentivirus and puromycin selection, PaTu8988 and CFPAC-1 cells were harvested for transwell invasion assay (H) and the quantitative data (I). n=3. **J-L**, The quantitative data of PaTu8988 and CFPAC-1 cells, which were infected of indicated lentivirus and puromycin selection, and harvested for clone formation assay (J), wound-healing assay (K) and transwell invasion assay (L). n=3. **M-N**, PaTu8988 cells transfected with indicated plasmids were injected to nude mice (5mice for each group) via caudal vein, after 30 days mice were sacrificed to evaluate the lung metastases, arrows indicate metastatic tumor foci in mouse lung and HE-stained sections of lung metastases were imaged under a microscope (M) and quantified (N). *n.s.*, not significant; *, *P* < 0.05; **,* P* < 0.01; ***, *P* < 0.001.

The inactivating mutations or copy number loss of the tumor suppressor gene,* BAP1*, have been observed in various malignancies, including pancreatic cancer. Previous research has demonstrated its role in the initiation of chronic pancreatitis driven carcinogenesis^11^. However, reduced BAP1 expression is also significantly correlated with poorer overall survival (OS) in TCGA-PAAD dataset (Fig. 1A). Comparative analysis of BAP1 expression between normal pancreatic ductal epithelial cells and tumor cells revealed a significant downregulation of BAP1 expression in the latter (Supplementary Fig. S1B). Of note, we conducted a comparative analysis of the prognosis between patients harboring homozygous or heterozygous deletions of BAP1 within the TCGA-PAAD dataset and those without such deletions. Intriguingly, despite the patients with BAP1 deletions demonstrating a trend towards a poorer prognosis, this observed difference failed to attain statistical significance (Supplementary Fig. S1A). This discrepancy may potentially be attributed to the limited sample size, which could have led to an uneven distribution or disparity in the number of patients harboring BAP1-deletion versus those with the wild-type BAP1 (BAP1-WT), thereby affecting the statistical power of the comparison. To gain a deeper understanding of the expression patterns of BAP1 in PDAC and its implications for prognosis, we conducted a comparative analysis of BAP1 expression in cancer tissues versus adjacent tissues within our internal cohort. Our observations revealed a notable downregulation of BAP1 expression in a subset of tumor tissues, which was associated with a significantly poorer prognosis for those patients (Fig. 1B-C), indicating BAP1 deficiency might also plays a significant role in the progression and metastasis of PDAC. To verify this hypothesis, we knocked out BAP1 using two independent single-guide RNA in two human pancreatic cancer cell lines with relatively high BAP1 expression, PaTu8988 and CFPAC-1 (Supplementary Fig. S1C, Supplementary Fig. S2A). We observed that the loss of BAP1 significantly promoted the growth, migration, and invasive capabilities of both cell lines examined via colony formation, wound healing and transwell assays (Fig. 1D-I). While the re-expression of BAP1 can counteract the impact of its knockout on the proliferative and metastatic capabilities of the cell lines examined (Fig. 1J-L, Supplementary Fig. S2B-E). We also observed consistent phenomena when we overexpressed BAP1 in PDAC cell lines with relatively low BAP1 expression, SW1990 and BxPC-3 (Supplementary Fig. S1C, Supplementary Fig. S2F-L). Considering the higher incidence of heterozygous loss of BAP1 in individuals diagnosed with pancreatic ductal adenocarcinoma, we established a shBAP1 cell line to more accurately replicate the pathological state observed in PDAC patients (Supplementary Fig. S1D). Consistent with our expectations, the shBAP1 cells exhibited augmented proliferative and invasive capabilities (Supplementary Fig. S1E-J). Furthermore, we validated the role of BAP1 using a PDAC lung metastasis mouse model. BAP1 knockout significantly increased the number of metastatic lesions, whereas re-expression of BAP1 suppressed this effect (Fig. 1M-N). Together, our current data elucidate the promotive role of BAP1 deficiency in the proliferation, migration and invasion of pancreatic cancer.

### BAP1 deficiency is correlated with NF-κB activation in PDAC patients

**Figure 2:**
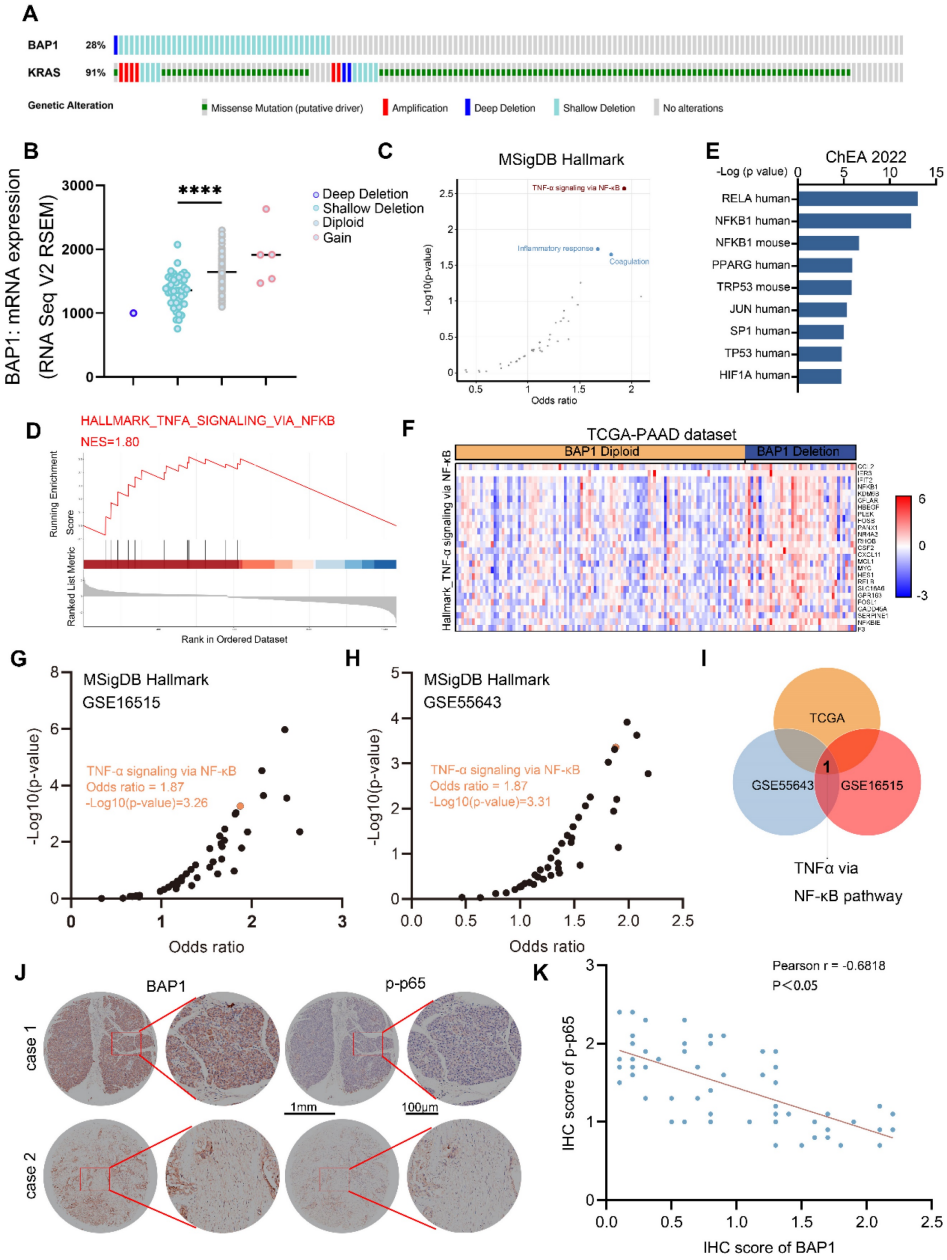
** BAP1 deficiency is correlated with NF-κB activation in PDAC patients. A**, Genetic alteration of indicated genes in TCGA-PAAD dataset. **B**, A scatter plot depicting the correlation between BAP1 copy number alterations and mRNA levels in the TCGA-PAAD dataset. **C**, A scatter plot showing the enriched pathway from differential genes between BAP1-Diploid and BAP1-Deletion patients in TCGA-PAAD dataset. **D**, GSEA output of the indicated pathways showing differential genes between BAP1-Diploid and BAP1-Deletion groups. **E**, Bar graph showing the top transcription factors profiled by differential genes between BAP1-Diploid and BAP1-Deletion groups from ChEA 2022 library using Enrichr. **F,** Heatmap of expression of DEGs between BAP1 Diploid and BAP1 Deletion in TNF-α via NF-κB pathway in TCGA-PAAD dataset.** G-H**, A scatter plot showing the enriched pathway from differential genes between BAP1 high and BAP1 low groups in GSE16515 (G) and GSE55643 (H) from MSigDB Hallmark library using Enrichr.** I**, Venn diagram shows the result of analysis of enriched pathway of differential genes between BAP1 high and BAP1 low groups in different dataset.** J-K**, Represent images of the IHC analysis of the indicated proteins on TMA(J) and correlation analysis(K). Spearman correlation coefficient and P value are shown. n=61. *n.s.*, not significant; *, *P* < 0.05; **,* P* < 0.01; ***, *P* < 0.001.

To further investigate the mechanisms by which BAP1 regulates pancreatic cancer progression, we analyzed the TCGA-PAAD dataset and found that 28% of the patients in this cohort harbor either homozygous or heterozygous deletions of BAP1, resulting in a significant downregulation of *BAP1* mRNA expression (Fig. 2A-B). Further analysis of the transcriptome data of the cohort indicated that NF-κB signaling was significantly upregulated in patients with BAP1 deletion (Fig. 2C-F). We further analyzed PDAC patient data from additional cohorts (GSE16515 and GSE55643) and also found a significant negative correlation between BAP1 expression and NF-κB signaling (Fig. 2G-H). Analysis of upregulated signaling pathways in patients with low BAP1 expression revealed that the NF-κB pathway is the only pathway consistently upregulated across all three datasets mentioned above (Fig. 2I). We further performed immunohistochemistry (IHC) analysis on the tissue microarrays from a group of PDAC patients, and found that the BAP1 protein expression was negatively correlated with the level of Ser536-phosphorylated p65 (p-p65(S536)), a marker of NF-κB activation (Fig. 2J-K). These patient data suggest that BAP1 might play a vital role in the regulation of NF-κB signaling.

### BAP1 represses the activation of NF-κB signaling

**Figure 3:**
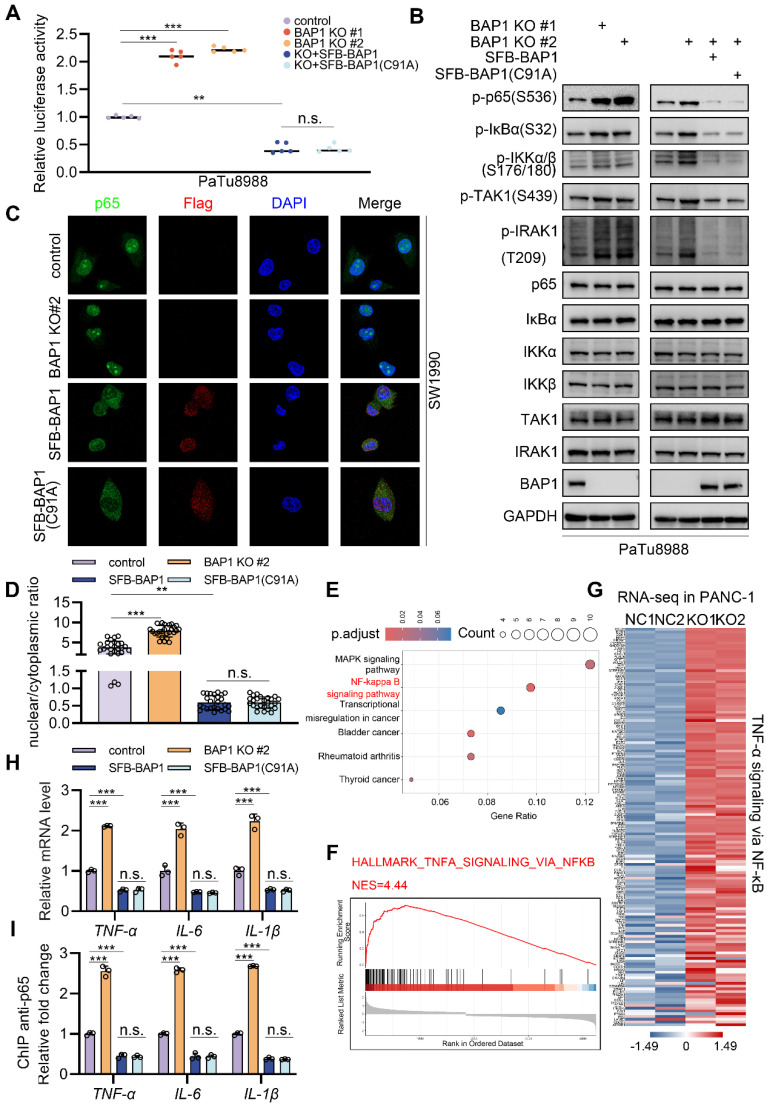
** BAP1 represses the activation of NF-κB signaling. A**, Luciferase reporter activities of p65 were assessed in PaTu8988 cells infected with indicated shRNAs and transfected with plasmids containing SFB-BAP1(WT) and SFB-BAP1(C91A). **B**, After the infection of indicated lentivirus and puromycin selection, PaTu8988 cells were harvested for western blot analysis.** C-D**, After the infection of indicated lentivirus and puromycin selection, SW1990 cells were harvested for immunocytochemistry (C) and bar plot shows the nuclear/cytoplasmic ratio in specific group (D).** E**, Kyoto Encyclopedia of Genes and Genomes (KEGG) analysis of DEG between NC and BAP1 KO group in GSE120078. **F**, GSEA output of the related pathway of DEG between NC and BAP1 KO group of GSE120078.** G**, Heatmap shows DEG between NC and BAP1 KO group in related pathway of GSE120078. **H-I**, PaTu8988 cells were infected with lentivirus expressing indicated shRNAs for 48 h. After a 48 hours puromycin selection, cells were harvested for RT-qPCR (H) and ChIP-qPCR (I) of indicated genes. *n.s.*, not significant; *, *P* < 0.05; **,* P* < 0.01; ***, *P* < 0.001.

To further verify whether BAP1 regulate NF-κB pathway, we assessed the activation level of NF-κB signaling via luciferase reporter assay. We found that BAP1 knock-out using single-guide RNA resulted in a prominent increase of NF-κB activity (Fig. 3A, Supplementary Fig. S3A). Interestingly, the re-expression of wild-type BAP1 or enzymatically inactive BAP1 (BAP1(C91A)) both significantly repressed the NF-κB activity (Fig. 3A, Supplementary Fig. S3A). Western blot analysis also revealed that BAP1 knock-out resulted in a significant increase in the phosphorylation levels of key proteins in NF-κB pathway, such as p-p65(S536), p-IRAK1(T209), p-IKKα and p-IKKβ(S176/180) (Fig. 3B), and the phenomenon could be rescued by either the re-expression of WT or C91A mutated BAP1 (Fig. 3B). In accordance with this observation, analogous phenomena were likewise noted in the shBAP1 cellular model (Supplementary Fig. S3B-C).

Immunocytochemistry (ICC) analysis in SW1990 also indicated that the over-expression of BAP1 or BAP1(C91A) significantly inhibited the nuclear localization level of p65 while knockdown of BAP1 exhibits opposite effects (Fig. 3C-D). Moreover, analysis of the transcriptome data of PANC-1 cells (GSE120078) indicated that the knock-out of BAP1 led to a dramatically activation of NF-κB signaling and a pronounced upregulation of its downstream genes (Fig. 3E-G, Supplementary Fig. S3D). We further validated this point via RT-qPCR and the ChIP-qPCR of p65 at the locus of indicated NF-κB downstream genes (Fig. 3H-I). These data further substantiate the observations we made in patient data, and also suggest that BAP1 may regulate the NF-κB pathway through a mechanism independent of its enzymatic activity.

### BAP1 interacts with IRAK1 in cytoplasm

**Figure 4:**
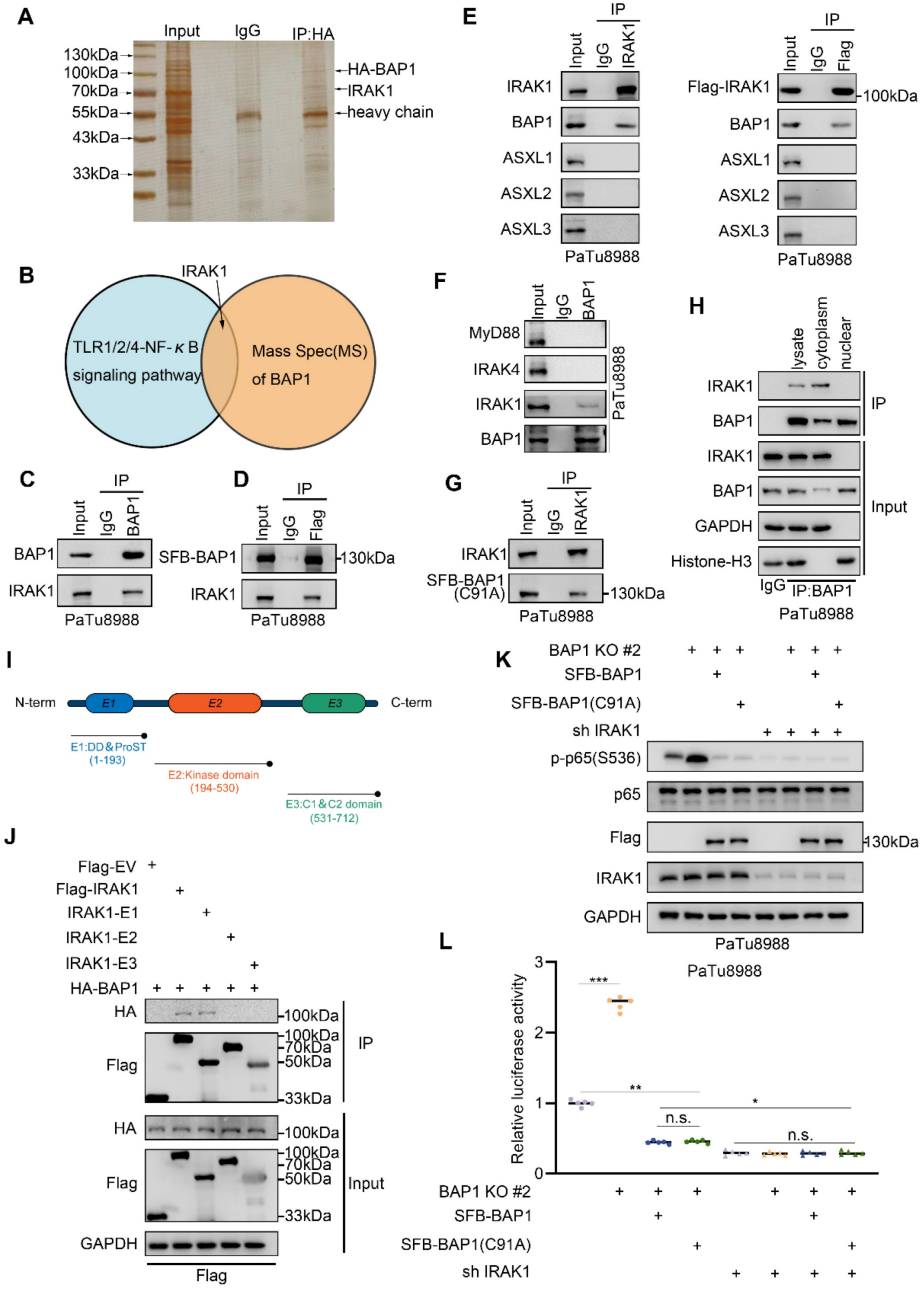
** BAP1 interacts with IRAK1 in cytoplasm. A**, Silver staining to verify the effectiveness of immunoprecipitation of HA-BAP1. **B**, Venn diagram shows the result of analysis of MS of BAP1 and specific pathway.** C-D**, PaTu8988 cells were harvested for reciprocal co-IP of BAP1(C) and after the infection of indicated plasmid, PaTu8988 cells were harvested for co-IP of Flag. **E**, PaTu8988 cells were harvested for reciprocal co-IP of IRAK1.** F**, PaTu8988 cells were harvested for reciprocal co-IP of BAP1.** G**, After the infection of indicated plasmid, PaTu8988 cells were harvested for co-IP of IRAK1.** H**, PaTu8988 cells were harvested for nuclear-cytoplasmic separation and for reciprocal co-IP of BAP1. **I**, Schematic diagram depicting a set of Flag-IRAK1 recombinant protein constructs. **J**, After the infection of indicated plasmid, 293T cells were harvested for co-IP of Flag. **K-L**, After the infection of indicated plasmid, PaTu8988 cells were harvested for western blot analysis (K) and luciferase reporter activities (L) of p65 were assessed in (K). n=5. *n.s.*, not significant; *, *P* < 0.05; **,* P* < 0.01; ***, *P* < 0.001.

BAP1 serves as the enzymatic unit of the PR-DUB complex, regulating chromatin conformation and gene transcription by deubiquitinating histone H2A^26^, ^27^. The enzymatic activity of BAP1 relies on its nuclear localization^28^. However, our observations suggest that BAP1 represses NF-κB activation independently of its enzymatic activity. Therefore, we hypothesize that BAP1 might regulate NF-κB through mechanisms beyond transcriptional repression via H2A. We overexpressed HA tagged BAP1 in 293T cells and identified its interacting proteins via co-immunoprecipitation and mass spectrometry analysis (Fig. 4A). Notably, IRAK1, the kinase responsible for IL-1 induced NF-κB activation, emerged as a robust interacting protein in the list (Fig. 4B). We validated the protein-protein interaction between BAP1 and IRAK1 through reciprocal co-immunoprecipitation (Co-IP) in endogenous and exogenous level (Fig. 4C-E). The activation of IL-1R/TLRs leads to the upregulation of NF-κB signaling mainly through MyD88-IRAK4-IRAK1 axis^29^. We were interested if BAP1 interacts with other important proteins of this axis. We demonstrated that ectopically over-expressed HA-BAP1 only interacted with IRAK1, but not MyD88 or IRAK4. Furthermore, ectopically overexpressed Flag-IRAK1 interacted solely with BAP1 and not with other members of the PR-DUB complex, ASXL1-3 (Fig. 4E-F), indicating a specific interaction between BAP1 and IRAK1. In alignment with our previous observations, both wild-type (WT) BAP1 and C91A mutated BAP1 are observed to bind to IRAK1 (Fig. 4G). The BAP1-IRAK1 interaction primarily takes place in the cytoplasm (Fig. 4H), where BAP1 is enzymatically inactive in the absence of ASXLs. We truncated IRAK1 into three segments based on its functional domains, as shown in the diagram (Fig. 4I). Co-IP assay showed that the DD & ProST domain in the N terminal of the IRAK1 interacts with BAP1 (Fig. 4J). More importantly, under the condition of IRAK1 knockdown, altering BAP1 no longer induces changes in p65 (S536) phosphorylation (Fig. 4K) and the transcriptional activity of NF-κB (Fig. 4L). Together, we identified the protein-protein interaction between BAP1 and IRAK1 in cytoplasm and elucidated that BAP1 might repress NF-κB through IRAK1.

### BAP1 inhibits the phosphorylation and the activation of IRAK1

**Figure 5:**
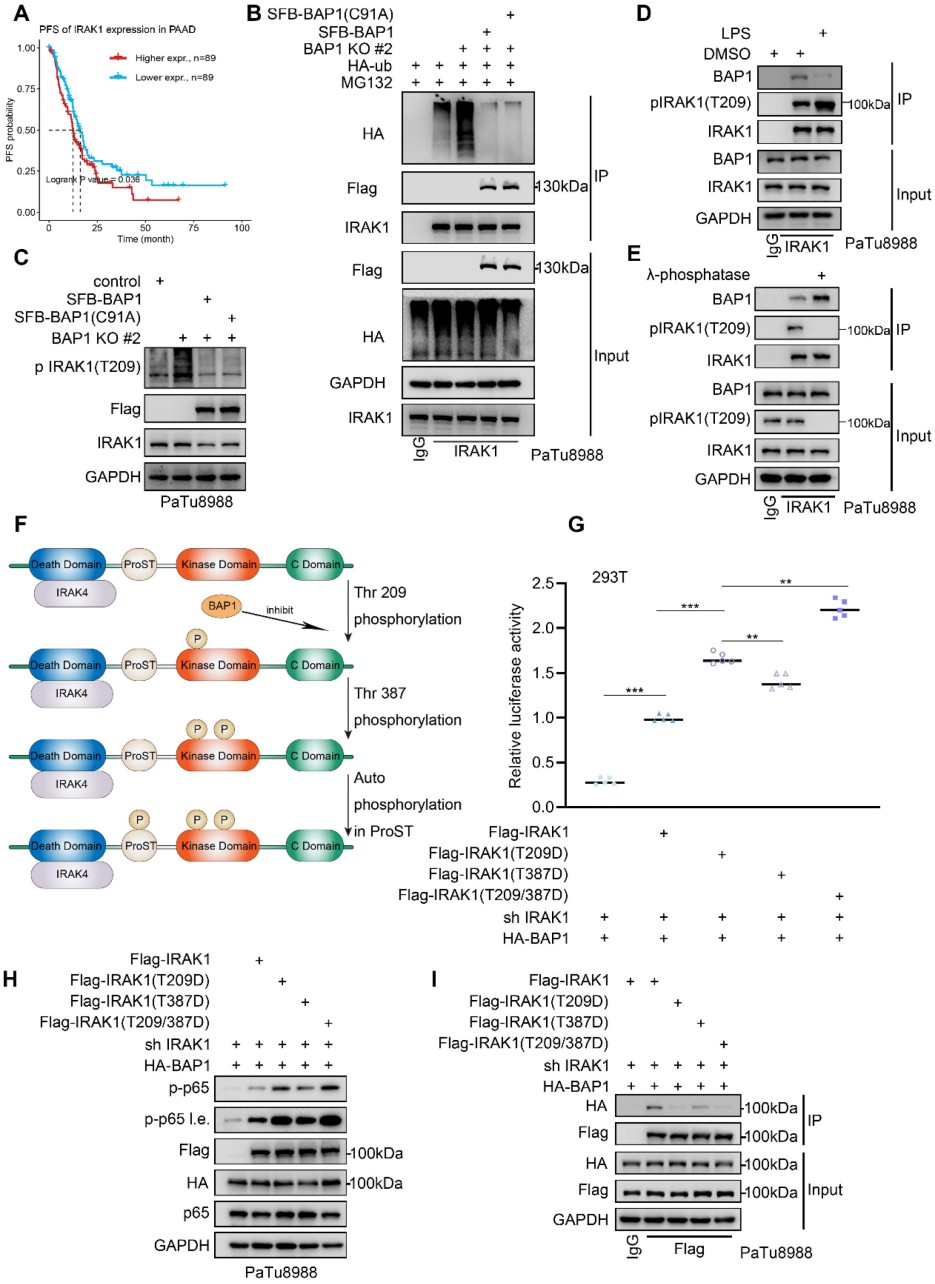
** BAP1 inhibits the phosphorylation and the activation of IRAK1. A**, Kaplan-Meier plots showing the PFS between IRAK1 high and low patients in TCGA PAAD dataset. **B**, PaTu8988 cells were infected with lentivirus expressing indicated shRNAs and plasmid transfected with HA-ub were harvested for ubiquitination assay.** C**, After the infection of indicated plasmid, PaTu8988 cells were harvested for western blot analysis.** D**, After the infection of indicated plasmid and lipopolysaccharide (LPS) treatment, PaTu8988 cells were harvested for western blot analysis.** E**, PaTu8988 cells were harvested for λ-phosphatase treatment and used for western blot analysis. **F**, Working model illustrating how IRAK1 phosphorylation and activating.** G**, Luciferase reporter activities of p65 was assessed in PaTu8988 cells infected with indicated shRNAs and transfected with plasmids containing Flag-IRAK1(WT), Flag-IRAK1(T209D), Flag-IRAK1(T389D) and Flag-IRAK1(T209/387D). n=5. **H-I**, After the infection of indicated plasmid, PaTu8988 cells were harvested for western blot analysis(H) and for co-IP of Flag(I). *n.s.*, not significant; *, *P* < 0.05; **,* P* < 0.01; ***, *P* < 0.001.

As a crucial mediator in the IL-1R mediated signaling pathway, IRAK1 exhibits a substantial upregulation in PDAC tumors when compared to adjacent peritumoral tissue and is significantly linked to a diminished progression-free survival (PFS) (Fig. 5A, Supplementary Fig. S4A). Upon the IL1R/TLR stimulation, IRAK1 is recruited and phosphorylated by IRAK4, leading to extensive autophosphorylation and ubiquitination. Subsequently, it dissociated from the Myddosome and activated the E3 ligase TRAF6, initiating the sequential activation of NF-κB signaling. Our previous data indicated that the knockout of BAP1 resulted in a significant upregulation of IRAK1(T209) phosphorylation (Fig. 3B). We were interested to verify whether BAP1 is involved in this process. The Co-IP assay indicated that the knockout of BAP1 significantly increased the ubiquitination level of IRAK1 while the mRNA level of IRAK1 did not show significant changes. (Fig. 5B, Supplementary Fig. 4B-D). However, overexpression of either the wild type or C91A mutated BAP1 equally inhibited the ubiquitination of IRAK1 (Fig. 5B, Supplementary Fig. 4E-F). Consistent with our previous observation, these data indicated that BAP1 might not influence the ubiquitination of IRAK1 via its enzymatic activity. Instead, BAP1 is more likely to affect the activation of IRAK1 and its subsequent ubiquitination. Thus, we proceeded to investigate the impact of BAP1 on IRAK1 T209 phosphorylation, the site that is phosphorylated at the onset of IRAK1 activation^30^. Similar to the pattern in ubiquitination assay, the knock-out of BAP1 significantly upregulated p-IRAK1(T209), while the expression of wild-type or catalytically inactive BAP1 equally inhibited the phosphorylation at this site (Fig. 5C, Supplementary Fig. 4G-H). We then tested if the phosphorylation status of IRAK1(T209) would affect BAP1-IRAK1 interaction. Lipopolysaccharide (LPS) treatment significantly increased the phosphorylation at IRAK1(T209) as expected, but also substantially inhibited the interaction between BAP1 and IRAK1 (Fig. 5D). Consistently, λ-phosphatase treatment prominently enhanced BAP1-IRAK1 interaction (Fig. 5E).

Previous studies indicated that the phosphorylation of IRAK-1 occurs through three sequential steps: initial phosphorylation at Thr209, followed by Thr387, and finally, hyperphosphorylation in the ProST region^31^ (Fig. 5F). To explore how BAP1 participate in the sequential phosphorylation of IRAK1, we construct several phosphor-mimic mutants of IRAK1 (T209D, T387D and T209D/T387D). By assessing NF-κB pathway activity through luciferase assays and p-p65(S536) detection, we found that all phospho-mimic mutants exhibited a greater ability to activate NF-κB compared to wild-type IRAK1. Notably, the double-site mutant (IRAK1(T209D/T387D)) demonstrated the strongest effect on NF-κB activation in the context of BAP1 expression (Fig. 5G-H). Consistently, Co-IP assay indicated that the binding affinity of BAP1 to these IRAK1 mutants was significantly reduced compared to wild-type IRAK1 accordingly (Fig. 5I). The interaction between BAP1 and IRAK1 is closely related to the phosphorylation status of IRAK1. BAP1 inhibits the sequential phosphorylation and activation of IRAK1 by preventing phosphorylation at the T209 site. Additionally, the phosphorylation status of IRAK1 itself competitively interferes with its binding to BAP1 (Fig. 5D-E).

### BAP1 inhibits the recruitment of IRAK1 by IRAK4

**Figure 6:**
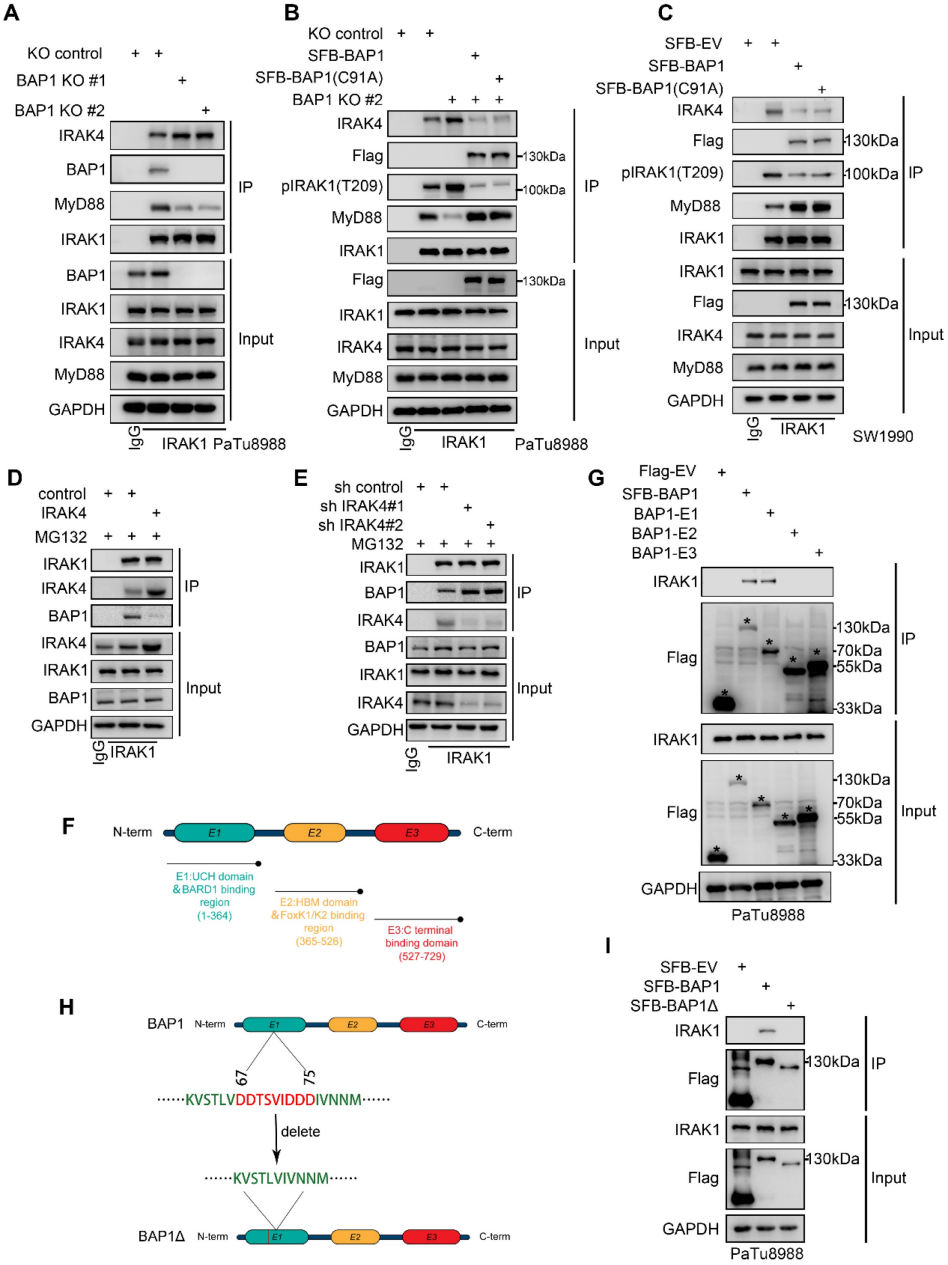
** BAP1 inhibits the recruitment of IRAK1 by IRAK4. A-C**, After the infection of indicated plasmid, PaTu8988(A-B) and SW1990(C) cells were harvested for co-IP of IRAK1. **D-E**, PaTu8988 cells were infected with lentivirus expressing indicated plasmid (D) and SiRNAs (E) were harvested for Co-IP of IRAK1.** F**, Schematic diagram depicting a set of Flag-BAP1 recombinant protein constructs. **G**, After the infection of indicated plasmid, PaTu8988 cells were harvested for co-IP of Flag.** H**, Schematic diagram depicting a set of Flag-BAP1 recombinant protein constructs. **I**, After the infection of indicated plasmid, PaTu8988 cells were harvested for co-IP of Flag.

BAP1 interacts with the death domain and Pro-ST domains of IRAK1, and the death domain of IRAK1 is responsible for the formation of the Myddosome (Fig. 4I-J). Subsequent to Myddosome formation, IRAK1 T209 undergoes phosphorylation by IRAK4. Our data suggests that BAP1-IRAK1 interaction inhibited IRAK1-T209 phosphorylation (Figure 5H-I). Thus, we were interested in whether BAP1 regulates IRAK1's interactions with other members of the Myddosome. Co-IP assay showed that BAP1 deletion significantly enhanced the binding of IRAK4 to IRAK1 and decreased the interaction between Myd88 and IRAK1 (Fig. 6A). Rescue experiment with either WT or C91A mutated BAP1 could compensate for the effect caused by BAP1 knock-out (Fig. 6B). Similarly, overexpression of BAP1 in the BAP1-low-expressing pancreatic cancer cell line SW1990 also resulted in a consistent phenomenon (Fig. 6C). After the phosphorylation on IRAK1 T209, IRAK1 initiates autophosphorylation, leading to the hyperphosphorylation of IRAK1 and the dissociation of MyD88 from the complex^31^, ^32^. Our data suggests that BAP1 loss enhances the binding of IRAK4 to IRAK1, resulting in the hyperphosphorylation of IRAK1, which, in turn, contributes to the reduced interaction between MyD88 and IRAK1. Considering that BAP1 can inhibit the above process without requiring enzymatic activity, we hypothesize that BAP1 likely binds to IRAK1 in a competitive manner with IRAK4.

To prove this, we silenced/overexpressed IRAK4 and detected whether the binding of BAP1 and IRAK1 was affected. Co-IP assay indicated that silenced IRAK4 increased the binding of BAP1 with IRAK1, while overexpression of IRAK4 inhibited the interaction between BAP1 and IRAK1 (Fig. 6D-E). Besides, we also truncated BAP1 into 3 constructs, and the following Co-IP experiment indicated that IRAK1 specifically binds with BAP1 E1 constructs, the UCH domain (Fig. 6F and G). Interestingly, the UCH domain contains a sequence of amino acids rich in negatively charged aspartic acid (^67^DDTSVIDDD^75^) (Fig. 6H). Since the interaction between BAP1 and IRAK1 is largely influenced by the phosphorylation status of IRAK1, we hypothesize that this negatively charged amino acid sequence on BAP1 may be crucial for the formation of the BAP1-IRAK1 complex. Thus, we built a BAP1∆ construction (deletion of ^67^DDTSVIDDD^75^) and observed that BAP1∆ no longer interacted with IRAK1 (Fig. 6H-I). Combining the above experiments, our data indicated that BAP1 competes with IRAK4 to bind with IRAK1. IRAK4 mediated IRAK1phosphorylation impedes IRAK1-BAP1 interaction. BAP1-IRAK1 interaction inhibited the sequential activation of IRAKs and the NF-κB signaling.

### BAP1-deficient PDAC confers the sensitivity to IRAK1/4 targeted inhibition

**Figure 7:**
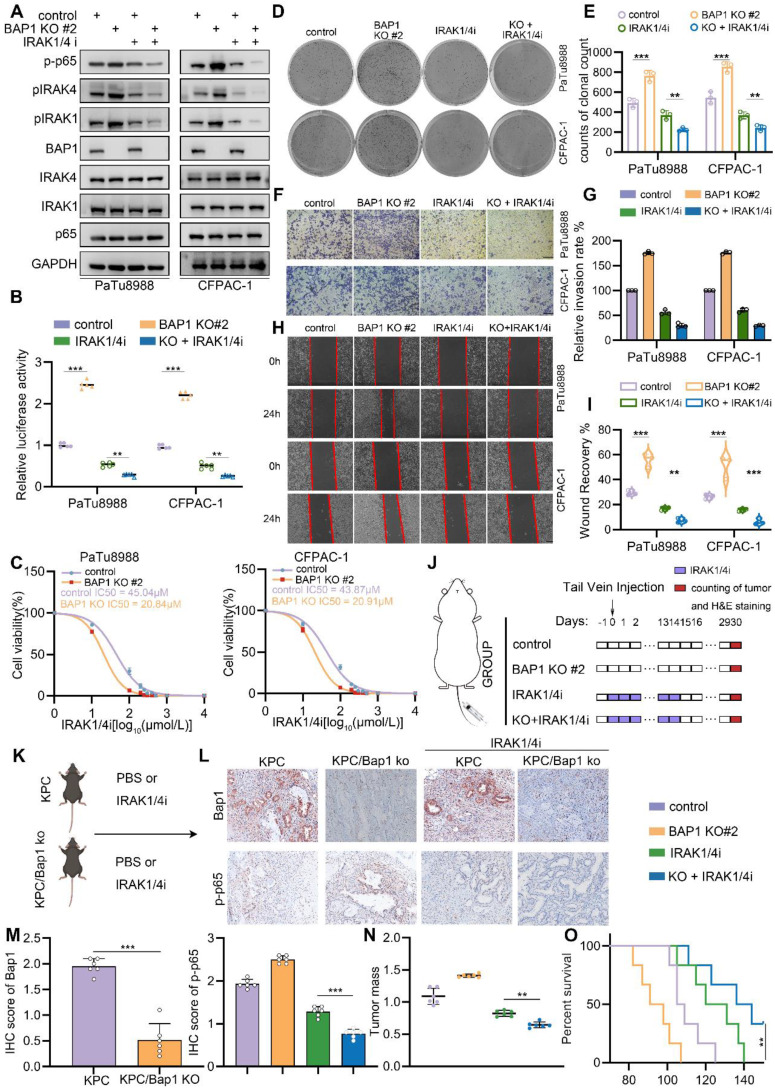
** BAP1-deficient PDAC confers the sensitivity to IRAK1/4 targeted inhibition. A,** PaTu8988 and CFPAC-1 cells were infected with lentivirus expressing indicated shRNAs and treated with IRAK1/4i were harvested for western blot analysis. **B,** Luciferase reporter activities of p65 were assessed in PaTu8988 and CFPAC-1 cells infected with indicated shRNAs and treated with IRAK1/4i. **C,** After 48 hours infection, the first set of cells was treated with different doses of IRAK1/4i for 48 hours and PaTu8988 and CFPAC-1 cells viability was measured by MTS assay. **D-E,** After indicated treatment, the clonogenicity of PaTu8988 and CFPAC-1 cells were measured (D) and quantified (E). n=3. **F-G,** After indicated treatment, PaTu8988 and CFPAC-1 cells were harvested for transwell invasion assay (F) and the quantitative data (G). n=3. **H-I,** After indicated treatment, PaTu8988 and CFPAC-1 cells were harvested for wound-healing assay (H) and the quantitative data (I). n=3. **J,** The diagram depicting the process of constructing lung metastasis models. **K,** The diagram depicting the process of genetically modified mice models. **L-M,** Representative images (L) and IHC score (M) of relevant proteins in KPC/KPC; Bap1KO mice. n = 6, two-tailed unpaired Student's t-test. **N,** The comparison of pancreas mass in the specified groups. n = 6,two-tailed unpaired t test.** O,** Survival analysis of the KPC/KPC; Bap1KO mice in specified groups. n.s., not significant; *, P < 0.05; **, P < 0.01; ***, P < 0.001.

Our results indicated that BAP1 represses NF-κB signaling through the regulation of IRAK1/4 complex. Then, targeting IRAK1/4 could be a potential therapeutic strategy to suppress enhancive tumorigenesis in BAP1 deficient pancreatic cancer. Furthermore, IRAK1 and IRAK4 share a conserved pocket structure and both IRAK1 and IRAK4 kinases participate in the same signaling pathway functionally, dual kinase inhibitors might provide more robust inhibition of NF-κB signaling compared to single-target inhibitor. Thus, we were interested in investigating the anti-tumoral effect of a dual IRAK1/4 inhibitor (named IRAK1/4i) in BAP1 deficient pancreatic cancer models. As expected, IRAK1/4i significantly suppressed activation of NF-κB signaling and the phosphorylation of IRAK1 (Fig. 7A-B). Interestingly, 50% inhibitory concentration (IC50) assessment indicated that BAP1 deletion sensitized pancreatic cancer cells to the treatment of IRAK1/4i (Fig. 7C). Furthermore, IRAK1/4i treatment considerably inhibited the clonogenicity, migration and invasion abilities of BAP1 deleted cells (Fig. 7D-I). As predicted, the shBAP1 cellular model exhibited a heightened susceptibility to inhibition by IRAK1/4i (Supplementary Fig. S5A-I). Consistent with the data *in vitro*, lung metastases of BAP1 knockout tumors were significantly suppressed by the treatment of IRAK1/4i compared to the control group (Fig. 7J, Supplementary Fig. S5J-K). We performed *in vivo* experiments utilizing genetically modified mice to investigate the impact of BAP1 knockout on the sensitivity to IRAKi^33^. Consistent with the findings observed in the lung metastasis model, the results indicated a significant inhibition of tumor growth in the BAP1 knockout group, when compared to the control group (Fig. 7K-M). Our findings above indicated that BAP1 deficient pancreatic cancer might be sensitive to the targeted inhibition of IRAK1/4.

## Discussion

*BAP1* maps to human chromosome 3p21.3, and the encoded BAP1 protein is found both in the nucleus and in the cytoplasm^34^. Previous studies have identified BAP1 as a tumor suppressor, particularly in mesotheliomas and metastatic uveal melanoma (UVM)^35^, ^36^, through its regulation of DNA repair, gene transcription, cell cycle progression, and various other cellular processes. However, these functions are mainly associated with nuclear BAP1^37^, and the role of cytoplasmic BAP1 remains poorly understood. Cytoplasm BAP1 has been described located at the endoplasmic reticulum (ER), and promoting apoptosis through regulating Ca^2+^ releasing^10^. Our current study identified cytoplasmic BAP1 competes with IRAK4 to bind IRAK1, and this process does not require BAP1's enzymatic activity. However, the biological functions of cytoplasmic BAP1 and the mechanisms governing its nuclear-cytoplasmic transport still require further investigation.

In prior investigations, BAP1 has been established to impede the transition from chronic pancreatitis to pancreatic cancer via the modulation of genomic stability^11^, ^38^. Furthermore, the deficiency of BAP1 triggers the ubiquitination and subsequent degradation of LATS2, ultimately culminating in the deregulation of the Hippo signaling pathway and the induction of pancreatic ductal adenocarcinoma in murine models^13^. These findings imply that the absence of BAP1 can augment cellular sensitivity to specific therapeutic interventions, thereby furnishing a theoretical rationale for therapies aimed at BAP1-associated cancers.

Additionally, our prior research has unveiled that BAP1 can modulate the activity of HSF1 through a non-enzymatic mechanism, ultimately impacting the responsiveness to immune checkpoint inhibitors^33^. However, these endeavors have primarily concentrated on nuclear BAP1. Given that BAP1 mutated in certain tumors may be sequestered in the cytoplasm, elucidating the role of cytoplasmic BAP1 is imperative for comprehending its physiological roles and pinpointing potential therapeutic avenues. In our current study, we discovered that cytoplasmic BAP1 inhibits the formation of the IRAK complex in an independent-enzymatic activity manner, unveiling a target that diverges from those previously reported. This discovery holds significant implications for advancing our understanding of the physiological functions of BAP1. Nonetheless, considering the phenomenon where BAP1 mutations in specific tumor types lead to its sequestration within the cytoplasm and subsequent loss of enzymatic functionality, there remains a pressing need for additional investigative efforts to delineate therapeutic strategies that specifically target cytosolic BAP1.In fact, the NF-κB pathway is overactivated in a significant proportion of pancreatic cancer patients. However, there are still various mechanisms that regulate the specific activation levels of NF-κB in the background of PDAC. The focus of our study is to investigate how the loss of BAP1 copy number, leading to a decrease in BAP1 expression, contributes to the further activation of NF-κB, compared to non-BAP1 loss PDAC. According to TCGA data, BAP1 heterozygous and homozygous deletions are observed in approximately 28% of PDAC patients (Figure 2B). Our study elucidates the negative correlation between BAP1 expression and NF-κB signaling in PDAC patient data and multiple PDAC models. Furthermore, the upregulation of NF-κB due to BAP1 loss has been observed and reported by several researchers^21^, ^39^, ^40^. However, the specific mechanisms underlying this phenomenon remain unexplained. Supported by these evidence, we further elucidated the specific mechanism by which BAP1 inhibits NF-κB signaling. We found that BAP1 downregulates NF-κB signaling by interacting with IRAK1 and inhibiting IRAK1-dependent NF-κB activation. Mechanistically, BAP1 directly binds to IRAK1, competitively inhibits the interaction between IRAK1 and IRAK4, significantly prevents phosphorylation at Thr209 and Thr378 of IRAK1, as well as the autophosphorylation of IRAK1, impeding the dissociation of IRAK1 from the Myddosome complex and the sequential activation NF-κB signaling.

Previous studies have demonstrated that IRAK1 undergoes phosphorylation and K63-linked ubiquitination during activation, both of which are essential for the initiation of downstream signaling pathways^30^, ^41^. In our study, we observed that BAP1 modulates IRAK1 phosphorylation, which in turn influences its ubiquitination status. Notably, this modification does not alter the overall protein levels of IRAK1. Interestingly, we found that this effect is independent of BAP1's enzymatic activity. Drawing on both existing literature and our findings, we propose that the absence of BAP1 leads to excessive phosphorylation of IRAK1, which consequently enhances K63-linked ubiquitination. This modification promotes the activation of TAK1 via TRAF6, ultimately resulting in the hyperactivation of the NF-κB pathway^30^, ^41^.

The IRAK family members are key molecules in the signaling cascade of the Toll/IL-1 receptor (TIR) family, which could incite the expression of inflammatory molecules within cancer cells^41^, ^42^ . It has been reported that constitutive IRAK4 activation drives resistance to checkpoint immunotherapy and chemotherapy and contributes to poor prognosis in PDAC^23^-^25^. However, current research on IRAK1/4 inhibitors mainly targets non-solid tumors, such as leukemia, suggesting that not all solid tumors are amenable to this treatment. Further research is needed to identify the potential patient populations that could benefit from IRAK1/4 inhibitors^43^. Here we demonstrated that BAP1 deficiency resulted in an abnormal activation of IRAKs and NF-κB signaling, thereby conferring the vulnerability to IRAK1/4 targeted inhibition, indicating PDAC patients with BAP1 deletion might benefit from treatment strategies including IRAK1/4 inhibition.

In summary, our study reveals a novel function of cytoplasmic BAP1 in suppressing NF-κB signaling by inhibiting the sequential activation of IRAK1/4. Furthermore, our findings indicate that targeting IRAK1/4 may offer a promising approach to counteract tumorigenesis associated with BAP1 deficiency in PDAC.

## Supplementary Material

Supplementary figures and tables.
