# Supplementary material for: BAP1 Represses Sequential Activation of IRAKs and NF-κB Signaling in Pancreatic Cancer
Source: Int J Biol Sci. 2025 Feb 18;21(5):1949–65. doi: 10.7150/ijbs.104977 (PMC11900812; doi:10.7150/ijbs.104977)
Supplement: Supplementary file 1 — Supplementary figures and tables. [file ijbsv21p1949s1.pdf]

Supplementary materials for

BAP1 Represses Sequential Activation of IRAKs and NF-κB Signaling in Pancreatic Cancer

Yuhan Zhao, Xueyi Liang, Ruozheng Wei, Feng Guo, Gengdu Qin, Haixin Yu, Jiaying Liu,

Wentao Xia, Shanmiao Gou, Heshui Wu, Yingke Zhou

The file includes:

- Supplementary figures and figure legends
- Supplementary Table S1. Information of recombinant DNA
- Supplementary Table S2. Sequence of primers and gene specific shRNAs & SiRNAs
- Supplementary Table S3. Information of antibodies
- Supplementary Table S4. Information of chemicals and kits

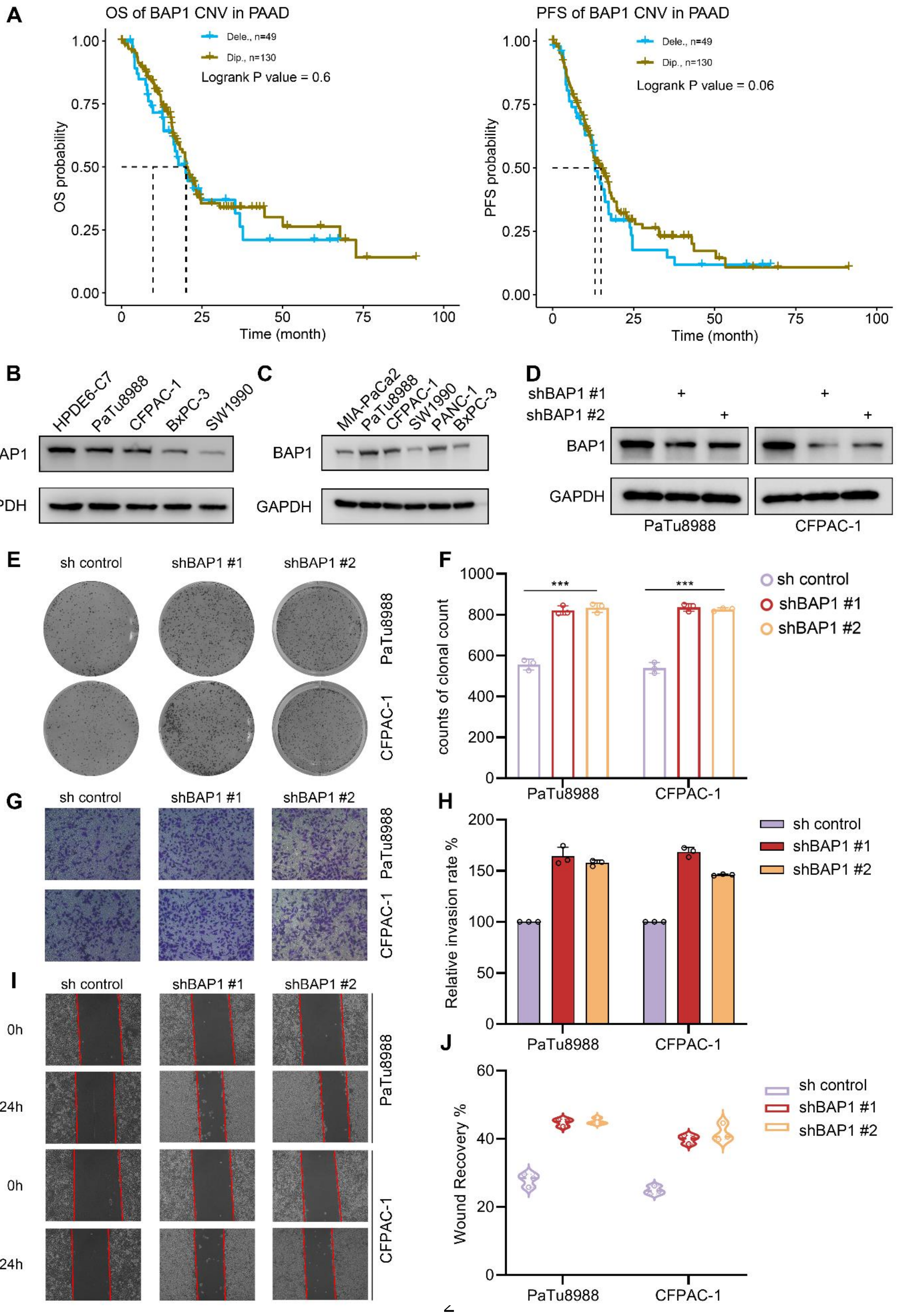

A
OS of BAP1 CNV in PAAD
Dele., n=49
Dip., n=130
Logrank P value = 0.6
OS probability
Time (month)
PFS of BAP1 CNV in PAAD
Dele., n=49
Dip., n=130
Logrank P value = 0.06
PFS probability
Time (month)
B
HPDE6-C7
PaTu8988
CFPAC-1
BxPC-3
SW1990
BAP1
GAPDH
C
MIA-PaCa2
PaTu8988
CFPAC-1
SW1990
PANC-1
BxPC-3
BAP1
GAPDH
D
shBAP1 #1
shBAP1 #2
BAP1
GAPDH
PaTu8988
CFPAC-1
E
sh control
shBAP1 #1
shBAP1 #2
PaTu8988
CFPAC-1
F
counts of clonal count
***
sh control
shBAP1 #1
shBAP1 #2
PaTu8988
CFPAC-1
G
sh control
shBAP1 #1
shBAP1 #2
PaTu8988
CFPAC-1
H
Relative invasion rate %
sh control
shBAP1 #1
shBAP1 #2
PaTu8988
CFPAC-1
I
sh control
shBAP1 #1
shBAP1 #2
0h
24h
PaTu8988
CFPAC-1
J
Wound Recovery %
sh control
shBAP1 #1
shBAP1 #2
PaTu8988
CFPAC-1

Supplementary Fig. S1 BAP1 loss promotes the proliferation, migration and invasion of PDAC cells (Supplementary data for Figure 1)

A, Kaplan-Meier plots showing the overall survival and progression free survival between BAP1 deletion and diploid patients in TCGA-PAAD dataset. **B**, Western blot analysis of BAP1 expression levels between normal pancreatic epithelial cells and the pancreatic cancer cell lines employed in this study. **C**, Western blot analysis of BAP1 in indicated pancreatic cancer cell lines. **D**, PaTu8988 and CFPAC-1 cells were infected with lentivirus expressing indicated shRNAs for 48 h. After a 48 hours puromycin selection, cells were harvested for western blot analysis. **E-F**, After the infection of indicated lentivirus and puromycin selection, 10 days after plate operation, the clonogenicity of PaTu8988 and CFPAC-1 cells were measured (E) and quantified (F). n=3. **G-H**, After the infection of indicated lentivirus and puromycin selection, PaTu8988 and CFPAC-1 cells were harvested for transwell invasion assay (G) and the quantitative data (H). n=3. **I-J**, After the infection of indicated lentivirus and puromycin selection, PaTu8988 and CFPAC-1 cells were harvested for wound-healing assay (I) and the quantitative data (J). n=3. *n.s.*, not significant; *, $P < 0.05$; **, $P < 0.01$; ***, $P < 0.001$.

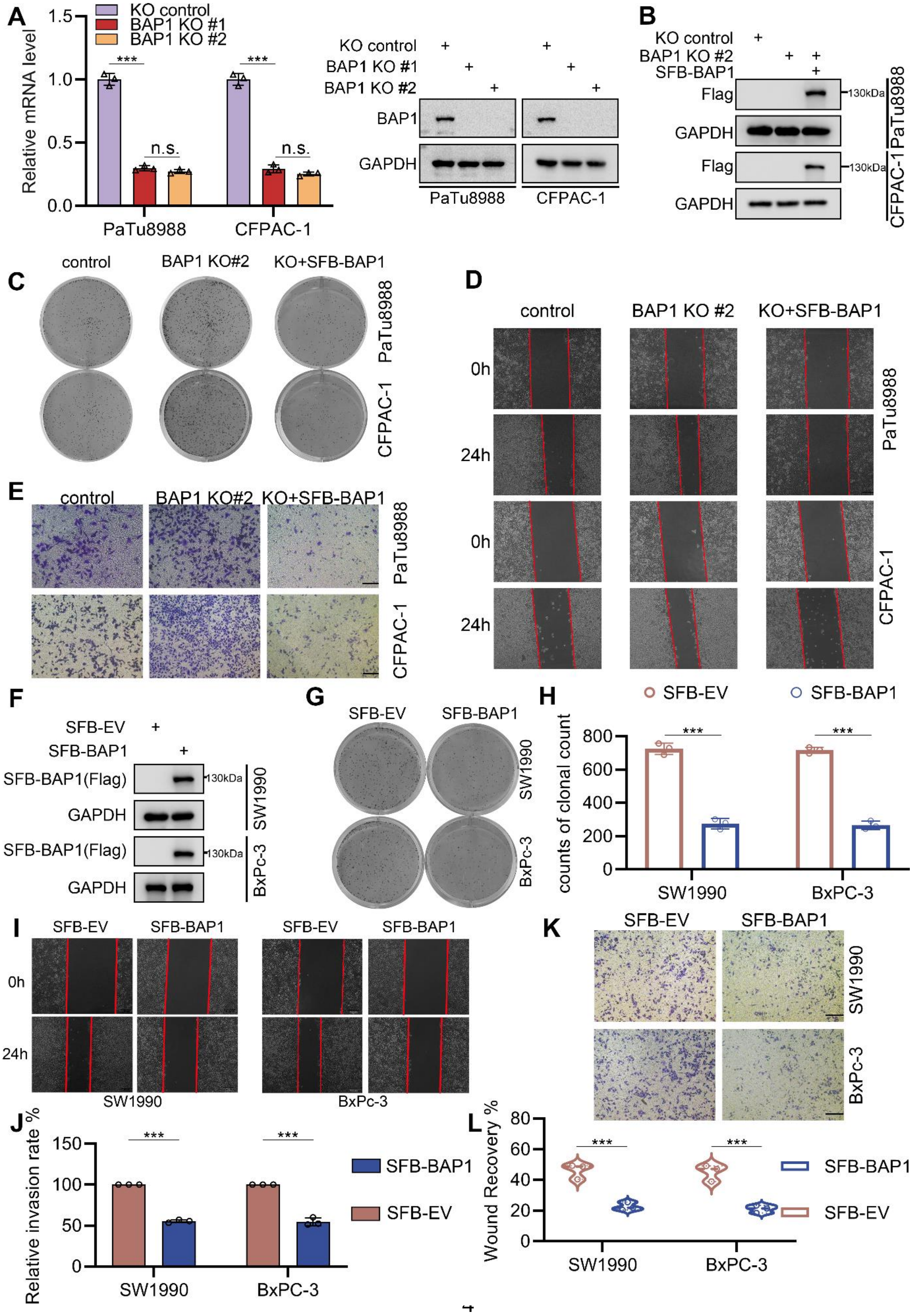
A
KO control
BAP1 KO #1
BAP1 KO #2
Relative mRNA level
***
n.s.
PaTu8988
CFPAC-1
BAP1
GAPDH
B
SFB-BAP1
Flag
130kDa
C
control
BAP1 KO#2
KO+SFB-BAP1
D
BAP1 KO #2
0h
24h
E
F
SFB-EV
SFB-BAP1(Flag)
SW1990
BxPc-3
G
H
counts of clonal count
BxPC-3
I
K
J
Relative invasion rate %
L
Wound Recovery %

Supplementary Fig. S2 BAP1 loss promotes the proliferation, migration and invasion of PDAC cells (Supplementary data for Figure 1)

A, PaTu8988 and CFPAC-1 cells were infected with lentivirus expressing indicated shRNAs for 48 h. After a 48 hours puromycin selection, cells were harvested for RT-qPCR (left) and western blot analysis (right). **B**, PaTu8988 and CFPAC-1 cells were infected with lentivirus expressing indicated shRNAs for 48 h. After a 48 hours puromycin selection, cells were harvested for western blot analysis. **C-E**, After the infection of indicated plasmids for 48h, PaTu8988 and CFPAC-1 cells were harvested for clone formation assay (C), wound-healing assay (D) and transwell invasion assay (E). n=3. **F**, After infection with lentivirus expressing indicated specific plasmids for 48h, SW1990 and BxPC-3 cells were harvested for western blot analysis. **G-H**, After the infection of indicated lentivirus and puromycin selection, 10 days after plate operation, the clonogenicity of SW1990 and BxPC-3 cells cells were measured (G) and quantified (H). n=3. **I-J**, After the infection of indicated lentivirus and puromycin selection, SW1990 and BxPC-3 cells were harvested for wound-healing assay (I) and the quantitative data (J). n=3. **K-L**, After the infection of indicated lentivirus and puromycin selection, SW1990 and BxPC-3 cells were harvested for transwell invasion assay (K) and the quantitative data (L). n=3. *n.s.*, not significant; *, $P < 0.05$; **, $P < 0.01$; ***, $P < 0.001$.

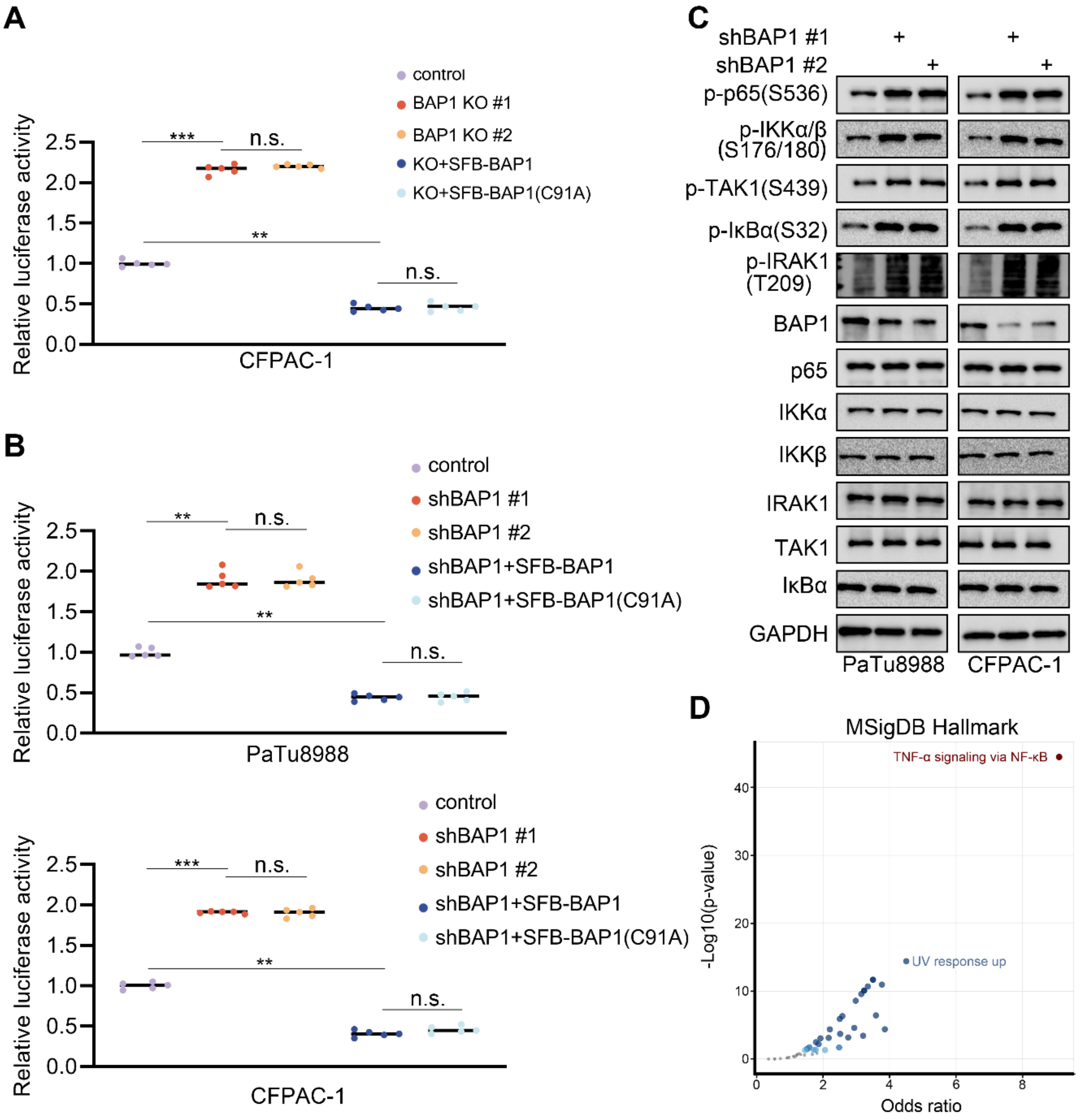
A
control
BAP1 KO #1
BAP1 KO #2
KO+SFB-BAP1
KO+SFB-BAP1(C91A)
Relative luciferase activity
2.5
2.0
1.5
1.0
0.5
0.0
***
n.s.
**
n.s.
CFPAC-1
B
control
shBAP1 #1
shBAP1 #2
shBAP1+SFB-BAP1
shBAP1+SFB-BAP1(C91A)
Relative luciferase activity
**
n.s.
**
n.s.
PaTu8988
***
n.s.
**
n.s.
CFPAC-1
C
shBAP1 #1
shBAP1 #2
+
+
p-p65(S536)
p-IKKα/β (S176/180)
p-TAK1(S439)
p-IκBα(S32)
p-IRAK1 (T209)
BAP1
p65
IKKα
IKKβ
IRAK1
TAK1
IκBα
GAPDH
PaTu8988
CFPAC-1
D
MSigDB Hallmark
TNF-α signaling via NF-κB
UV response up
-Log10(p-value)
40
30
20
10
0
0
2
4
6
8
Odds ratio

Supplementary Fig. S3 BAP1 represses the activation of NF-κB pathway via inactivation of IRAK1

A-B, Luciferase reporter activities of p65 were assessed in CFPAC-1 cells infected with indicated shRNAs and transfected with plasmids containing SFB-BAP1(WT) and SFB-sBAP1(C91A). n=5. **C**, After the infection of indicated lentivirus and puromycin selection, PaTu8988 and CFPAC-1 cells were harvested for western blot analysis. **D**, A scatter plot showing the enriched pathway from differential genes between NC and BAP1 KO groups from MSigDB Hallmark library using GSEA analysis. *n.s.*, not significant; *, $P < 0.05$; **, $P < 0.01$; ***, $P < 0.001$.

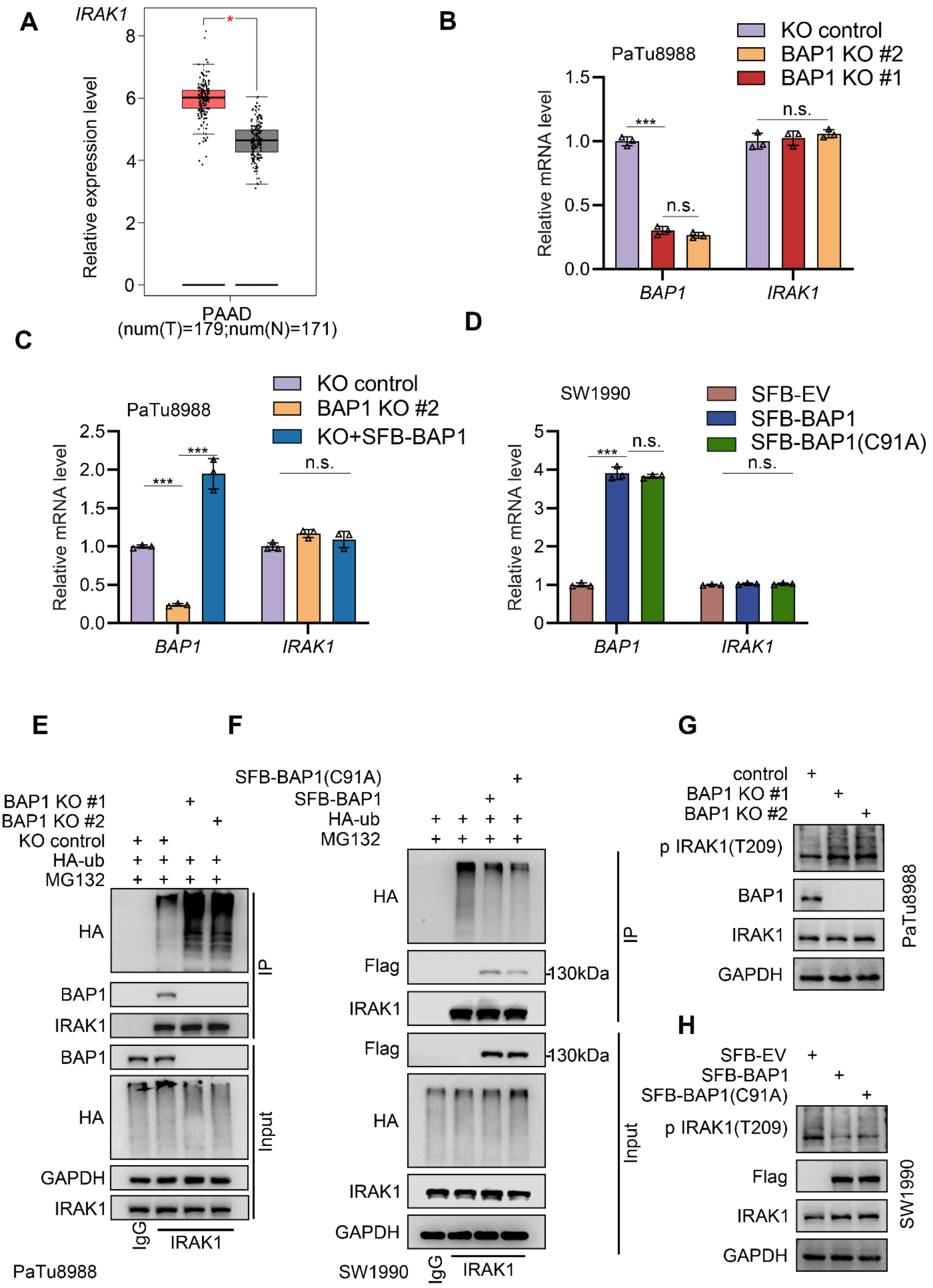
A
IRAK1
Relative expression level
8
6
4
2
0
*
PAAD
(num(T)=179;num(N)=171)
B
PaTu8988
KO control
BAP1 KO #2
BAP1 KO #1
Relative mRNA level
1.5
1.0
0.5
0.0
***
n.s.
n.s.
BAP1
IRAK1
C
PaTu8988
KO control
BAP1 KO #2
KO+SFB-BAP1
Relative mRNA level
2.5
2.0
1.5
1.0
0.5
0.0
***
***
n.s.
BAP1
IRAK1
D
SW1990
SFB-EV
SFB-BAP1
SFB-BAP1(C91A)
Relative mRNA level
5
4
3
2
1
0
***
n.s.
n.s.
BAP1
IRAK1
E
BAP1 KO #1
BAP1 KO #2
KO control
HA-ub
MG132
HA
BAP1
IRAK1
IP
BAP1
HA
GAPDH
IRAK1
Input
IgG
IRAK1
PaTu8988
F
SFB-BAP1(C91A)
SFB-BAP1
HA-ub
MG132
HA
Flag
130kDa
IRAK1
IP
Flag
130kDa
HA
IRAK1
GAPDH
Input
SW1990
IgG
IRAK1
G
control
BAP1 KO #1
BAP1 KO #2
p IRAK1(T209)
BAP1
IRAK1
GAPDH
PaTu8988
H
SFB-EV
SFB-BAP1
SFB-BAP1(C91A)
p IRAK1(T209)
Flag
IRAK1
GAPDH
SW1990

Supplementary Fig. S4 BAP1 represses the activation of IRAK1

A, A box plot depicting the correlation between BAP1 copy number alterations and mRNA levels in the TCGA-PAAD dataset. **B-D**, After the infection of indicated lentivirus and puromycin selection, PaTu8988 and SW1990 cells were harvested for RT-qPCR. n=3. **E-F**, PaTu8988 (E) and SW1990 (F) cells were infected with lentivirus expressing indicated shRNAs and plasmid transfected with HA-ub were harvested for ubiquitination assay. **G-H**, After the infection of indicated plasmid, PaTu8988 (G) and SW1990 (H) cells were harvested for western blot analysis. *n.s.*, not significant; *, $P < 0.05$; **, $P < 0.01$; ***, $P < 0.001$.

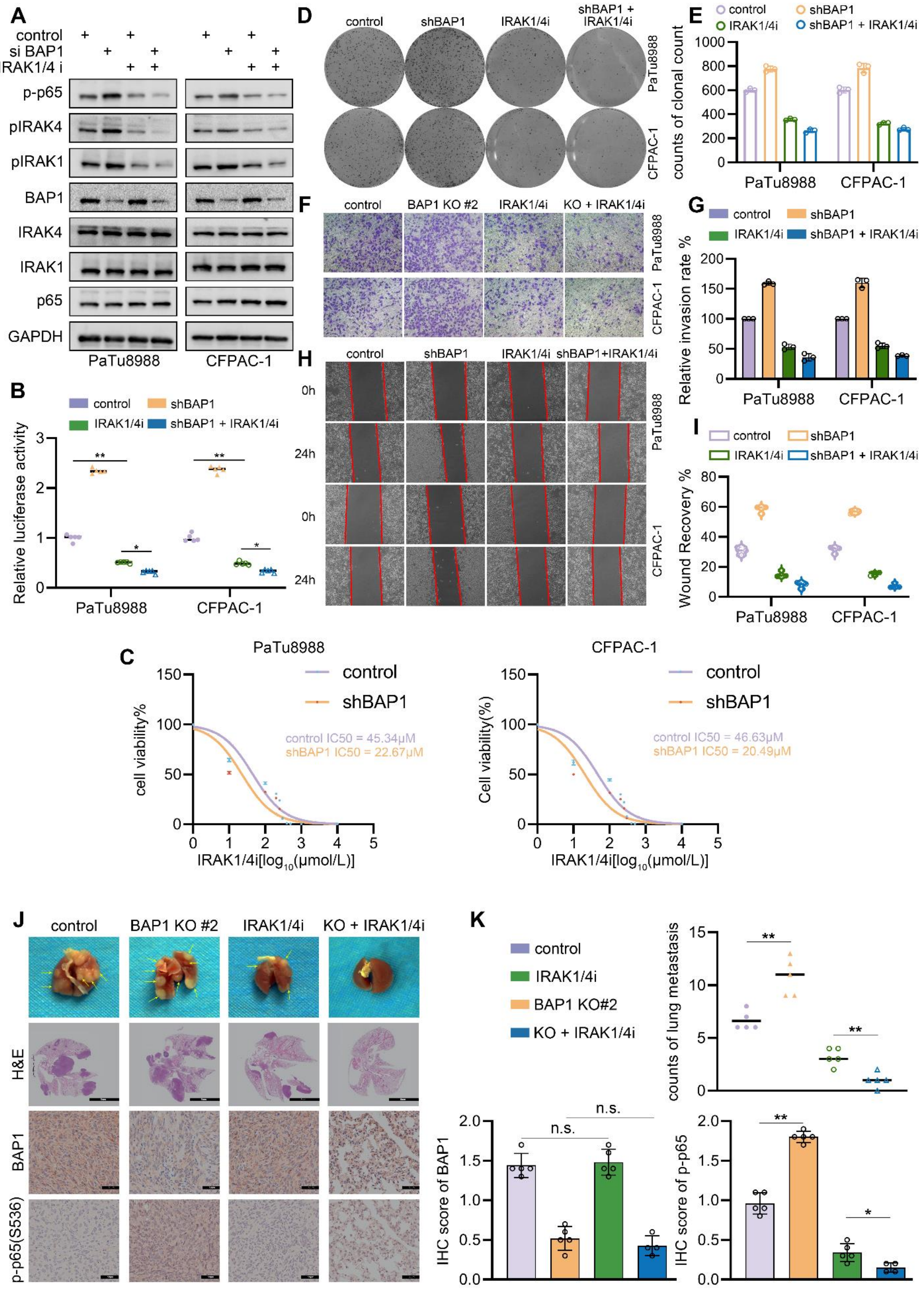

A
control
si BAP1
IRAK1/4 i
p-p65
pIRAK4
pIRAK1
BAP1
IRAK4
IRAK1
p65
GAPDH
PaTu8988
CFPAC-1
B
control
shBAP1
IRAK1/4i
shBAP1 + IRAK1/4i
Relative luciferase activity
PaTu8988
CFPAC-1
C
PaTu8988
CFPAC-1
control
shBAP1
control IC50 = 45.34μM
shBAP1 IC50 = 22.67μM
control IC50 = 46.63μM
shBAP1 IC50 = 20.49μM
cell viability%
Cell viability(%)
IRAK1/4i[log10(μmol/L)]
D
control
shBAP1
IRAK1/4i
shBAP1 + IRAK1/4i
PaTu8988
CFPAC-1
E
counts of clonal count
F
control
BAP1 KO #2
IRAK1/4i
KO + IRAK1/4i
G
Relative invasion rate %
H
shBAP1+IRAK1/4i
0h
24h
I
Wound Recovery %
J
control
BAP1 KO #2
IRAK1/4i
KO + IRAK1/4i
H&E
BAP1
p-p65(S536)
K
control
IRAK1/4i
BAP1 KO#2
KO + IRAK1/4i
counts of lung metastasis
IHC score of BAP1
IHC score of p-p65
n.s.

Supplementary Fig. S5 BAP1-deficient PDAC confers the sensitivity to IRAK1/4 targeted inhibition

A, PaTu8988 and CFPAC-1 cells were infected with lentivirus expressing indicated shRNAs and treated with IRAK1/4i were harvested for western blot analysis. **B,** Luciferase reporter activities of p65 were assessed in PaTu8988 and CFPAC-1 cells infected with indicated shRNAs and treated with IRAK1/4i. **C,** After 48 hours infection, the first set of cells was treated with different doses of IRAK1/4i for 48 hours and PaTu8988 and CFPAC-1 cells viability was measured by MTS assay. **D-E,** After indicated treatment, the clonogenicity of PaTu8988 and CFPAC-1 cells were measured (D) and quantified (E). n=3. **F-G,** After indicated treatment, PaTu8988 and CFPAC-1 cells were harvested for transwell invasion assay (F) and the quantitative data (G). n=3. **H-I,** After indicated treatment, PaTu8988 and CFPAC-1 cells were harvested for wound-healing assay (H) and the quantitative data (I). n=3. **J-K,** PaTu8988 cells infected with sh-control or sh-BAP1 were intravenously injected into nude mice through caudal vein, and were treated with IRAK1/4i or PBS. The tumors were excised 30 days after injection for photograph (J top), H&E staining (J middle), and IHC staining (J bottom), while visible lung metastasis nodules and IHC score were quantified (K). *n.s.*, not significant; *, $P < 0.05$; **, $P < 0.01$; ***, $P < 0.001$.

Supplementary Table S1. Information of recombinant DNA

**Recombinant DNA**	**Source**
Flag-IRAK1	GeneChem
Flag-IRAK4	GeneChem
SFB(S protein-FLAG-Streptavidin binding peptide, SFB)-BAP1/SFB-BAP1(C91A)/SFB-BAP1(C91S)/SFB-EV/HA-EV/HA-BAP1 were a gift of Huang's lab (Mayo Clinic); Flag-IRAK1(T209D)/ Flag-IRAK1(T387D)/ Flag-IRAK1(T209/387D) and constructs of BAP1/IRAK1 were provided by OBiO Technology (Shanghai).	

Supplementary Table S2. Sequence of primers and gene specific shRNAs & SiRNAs

**Gene**	**Usage**	**Forward**	**Reverse**
GAPDH	RT-qPCR	GTCAACGGATTTGGTCGTAT	GAACATGTAAACCATGTAGTTGA
BAP1	RT-qPCR	GACCCAGGCCTCTTCACC	AGTCCTTCATGCGACTCAGG
IRAK1	RT-qPCR	GACCCTGTCTCTGCCAAAAA	CAGGCTGGAGTGCAGTCATA
TNF-α	RT-qPCR	GAGGCCAAGCCCTGGTATG	CGGGCCGATTGATCTCAGC
IL-6	RT-qPCR	ACTCACCTCTTCAGAACGAATTG	CCATCTTTGGAAGGTTCAGGTTG
IL-1β	RT-qPCR	ATGATGGCTTATTACAGTGGCAA	GTCGGAGATTCGTAGCTGGA

TNF-α *promoter*	ChIP-qPCR	TGGGGGTAGGGTTAGTACCG	CTACAGGCTTGTCACTCGGG
IL-6 *promoter*	ChIP-qPCR	GCTCCCTACACACATGCCTT	CCTTCCCTGTGCATGGTGAT
IL-1β *promoter*	ChIP-qPCR	ACTACCAGTCCTGACTCCCT	GCCACCGAAGACTATCCTCC

**shRNAs**	**Sequence**
shIRAK1	GTCAAAGTTCTCATGGTCAAA
shIRAK4-1	GCTGTGCTTCTCTGACAGGTA
shIRAK4-2	CCCAGACATTAAGAAGGTTCA
SgBAP1-1/2 and shBAP1-1/2 were a gift of Huang's lab (Mayo Clinic).	

Supplementary Table S3. Information of antibodies

Antibodies	Source	Identifier	Working dilution
Rabbit polyclonal anti-HA-tag Antibody	Proteintech	Cat # 51064-2-AP; RRID: AB_11042321	1:1000
Rabbit polyclonal anti-GAPDH Antibody	Proteintech	Cat #10494-1-AP; RRID: AB_2263076	1:3000
Rabbit polyclonal anti-BAP1 Antibody	Proteintech	Cat # 10398-1-AP; RRID: AB_2180460	1:1000
Rabbit Polyclonal anti-Flag-tag Antibody	Proteintech	Cat # 20543-1-AP; RRID: AB_11232216	1:1000
IRAK1 Polyclonal antibody	Proteintech	Cat # 10478-2-AP; RRID: AB_2126032	1:1000
IRAK4 Polyclonal antibody	Proteintech	Cat # 18221-1-AP; RRID: AB_2878519	1:1000
MYD88 Polyclonal antibody	Proteintech	Cat # 23230-1-AP; RRID: AB_2879236	1:5000
NF-κB p65 Recombinant antibody	Proteintech	Cat # 80979-1-RR; RRID: AB_2918923	1:5000
NF-κB p65 (D14E12) XP® Rabbit mAb	Cell Signaling Technology	Cat #8242; RRID: AB_10859369	1:1000
Phospho-NF-κB p65 (Ser536) (93H1) Rabbit mAb	Cell Signaling Technology	Cat #3033; RRID: AB_331284	1:1000
IKKα (3G12) Mouse mAb	Cell Signaling Technology	Cat # 11930; RRID: AB_2687618	1:1000

IKKβ (D30C6) Rabbit mAb	Cell Signaling Technology	Cat #; 8943<br>RRID: AB_11024092	1:1000
IκBα (L35A5) Mouse mAb (Amino-terminal Antigen)	Cell Signaling Technology	Cat #4814;<br>RRID: AB_390781	1:1000
Phospho-IKKα/β (Ser176/180) (16A6) Rabbit mAb	Cell Signaling Technology	Cat #2697;<br>RRID: AB_2079382	1:1000
Phospho-IκBα (Ser32) (14D4) Rabbit mAb	Cell Signaling Technology	Cat #2859;<br>RRID: AB_561111	1:1000
Phospho-IRAK1 (Thr209) Antibody	Cell Signaling Technology	Cat #12756;<br>RRID: AB_3532146	1:500
Phospho-IRAK4 (Thr345/Ser346) (D6D7) Rabbit mAb	Cell Signaling Technology	Cat # 11927;<br>RRID: AB_2797770	1:1000
ASXL1 (D1B6V) Rabbit mAb	Cell Signaling Technology	Cat #52519;<br>RRID: AB_2799415	1:1000
ASXL2 (E6Z3X) Rabbit mAb	Cell Signaling Technology	Cat # 71257;<br>RRID: AB_3532147	1:1000
ASXL3 Rabbit Antibody	Abmart	Cat # PJP06138;<br>RRID: AB_3532148	1:1000
LATS2-Specific Polyclonal antibody	Proteintech	Cat #20276-1-AP;<br>RRID: AB_10697657	1:1000
HRP Conjugated AffiniPure Goat Anti-Mouse IgG (H+L)	Boster Biological Technology	Cat#BA1050;<br>RRID: AB_2904507	1:4000
Mouse anti-rabbit IgG (Conformation specific)	Cell Signaling Technology	Cat# 5127;<br>RRID: AB_10892860	1:4000

monoclonal antibody (HRP conjugate)			

Supplementary Table S4. Information of chemicals and kits

Chemicals	Source	Identifier
DMSO	Sorlabio	D8371
Lipo8000™ Transfection Reagent	Beyotime	C0533
Opti-MEM	Gibco	11058021
Polybrene	Beyotime	C0351
Puromycin Dihydrochloride	Beyotime	ST551
DMEM	Gibco	11965092
PBS	Gibco	20012050
Fetal Bovein Serum （FBS）	Gibco	10099141
RIPA lysis buffer	Beyotime	P0013B
TRIzol reagent	Thermo Fisher Scientific	15596018

PMSF protease inhibitor	Beyotime	P1046
Protein A/G agarose beads	Beyotime	P2055
Phosphatase inhibitor A	Beyotime	P1082
Phosphatase inhibitor B	Beyotime	P1087
Lookout Mycoplasma PCR Detection Kit	Sigma-Aldrich	MP0035
PrimeScript™ RT Reagent Kit	Takara Bio Inc.	RR037A
TB Green™ Fast qPCR Mix PCR Kit	Takara Bio Inc.	RR430A
BCA Protein Assay Kit	Beyotime	P0012S
Tween-20	Biosharp	BS100
TBS	Servicebio	G0001
Triton X-100	Biosharp	BS084
IRAK1/4 inhibitorI	Sigma	I5409
NF-κb Luciferase Reporter Lentivirus	Creative Biogene	LVG00115Z
